# Practice Facilitation to Address Unhealthy Alcohol Use in Primary Care: A Cluster Randomized Clinical Trial

**DOI:** 10.1001/jamahealthforum.2024.2371

**Published:** 2024-08-09

**Authors:** Alison N. Huffstetler, Gabriela Villalobos, Ben Webel, Michelle S. Rockwell, Adam Funk, Roy T. Sabo, John W. Epling, E. Marshall Brooks, Jacqueline B. Britz, Beth A. Bortz, Dace S. Svikis, Albert J. Arias, Ryan Nguyen Tran, Alex H. Krist

**Affiliations:** 1 Department of Family Medicine and Population Health, Virginia Commonwealth University, Richmond; 2 Department of Family and Community Medicine, Virginia Tech Carilion School of Medicine, Roanoke; 3 Department of Biostatistics, Virginia Commonwealth University, Richmond; 4 Virginia Center For Health Innovation, Richmond; 5 Department of Psychology, Virginia Commonwealth University, Richmond; 6 Department of Psychiatry, Virginia Commonwealth University, Richmond; 7 Inova Fairfax Family Practice, Fairfax, Virginia

## Abstract

**Importance:**

Unhealthy alcohol use (UAU) is the fourth most preventable cause of death in the US. The US Preventive Services Task Force recommends that primary care clinicians routinely screen all adults 18 years and older for UAU; however, this preventive service is poorly implemented.

**Objective:**

To determine if practice facilitation improved delivery of the recommended care for UAU compared to usual care.

**Design, Setting, and Participants:**

This practice-level cluster randomized clinical trial was conducted across diverse and representative primary care practices throughout Virginia. A total of 76 primary care practices enrolled between October 2019 and January 2023.

**Intervention:**

Practices received immediate (intervention) or 6-month delayed (control) practice facilitation, which included tailored educational sessions, workflow management, and tools for addressing UAU.

**Main Outcomes and Measures:**

Outcomes included the increase in recommended screening for UAU, brief interventions, referral for counseling, and medication treatment. Data were collected via medical record review (structured and free text data) and transcripts of practice facilitator sessions and exits interviews.

**Results:**

Of the 76 primary care practices enrolled, 32 were randomized to intervention and 35 to control; 11 789 patients (mean [SD] age, 50.1 [16.3] years; 61.1% women) were randomly selected for analysis, with patient demographics similar to Virginia at large. From baseline to 6 months after intervention, screening with a validated instrument increased from 2.1% (95% CI, 0.5%-8.4%) to 35.5% (95% CI, 11.5%-69.9%) in the intervention group compared to 0.4% (95% CI, 0.1%-1.8%) to 1.4% (95% CI, 0.3%-5.8%) in the control group (*P* < .001). Brief office-based interventions for the intervention group increased from 26.2% (95% CI, 14.2%-45.8%) to 62.6% (95% CI, 43.6%-78.3%) vs 45.5% (95% CI, 28.0%-64.1%) to 55.1% (95% CI, 36.5%-72.3%) in the control group (*P* = .008). Identification of UAU, referral for counseling, and medication treatment had similar changes for both groups. Qualitative analyses of transcripts revealed that few clinicians understood the preventive service prior to practice facilitation, but at the end most felt much more competent and confident with screening and brief intervention for UAU.

**Conclusions and Relevance:**

This cluster randomized clinical trial demonstrated that practice facilitation can help primary care practices to better implement screening and counseling for UAU into their routine workflow. Effective primary care practice implementation interventions such as this can have a profound effect on the health of communities. Given the number of people that the participating practices care for, this intervention resulted in an additional 114 604 patients being screened annually for UAU who would not have been otherwise.

**Trial Registration:**

ClinicalTrials.gov Identifier: NCT04248023

## Key Points

**Question:**

Did tailored practice facilitation improve rates of recommended screening and management of unhealthy alcohol use (UAU) in primary care practices?

**Findings:**

In this cluster randomized clinical trial of 76 primary care practices, use of recommended screening instruments for UAU increased by 41.6% 6 months after intervention practices received tailored education, tools, and workflows, and brief clinic-based counseling also improved by 35.6%.

**Meaning:**

Primary care practices that are willing to address workflow and approach to screening and counseling for UAU can dramatically increase their delivery of this recommended preventive service, which will improve health outcomes for patients.

## Teaser

This cluster randomized clinical trial aims to determine if tailored practice facilitation improved rates of recommended screening and management of unhealthy alcohol use compared to usual care.

## Introduction

Unhealthy alcohol use (UAU) is the fourth leading cause of preventable death in the US.^1^ UAU includes a spectrum from risky drinking (drinking more than the recommended daily or weekly limits and binge drinking) to alcohol use disorder (AUD; inability to stop or control drinking alcohol).^2,3^ The US Preventive Services Task Force (USPSTF) recommends that primary care clinicians screen all adults 18 years and older for UAU with brief screening instruments like the Alcohol Use Disorders Identification Test-Consumption (AUDIT-C) or the Single Alcohol Screening Question (SASQ).^4,5^

Approximately 21% of adults engage in UAU.^6^ Yet, primary care practices have poorly implemented the USPSTF recommendation to screen and counsel patients.^7,8^ If clinicians do not use the recommended screening instruments, only 1% to 10% of patients with UAU are identified.^9,10,11,12^ When people screen positive, clinicians should obtain a more detailed history to differentiate risky drinking from AUD. All people with UAU should receive initial counseling, which can be delivered by primary care and takes 2 to 90 minutes. It includes normalized personal feedback or motivational interviewing delivered over 1 or multiple visits.^13^ People with AUD should also receive more intense psychosocial interventions such as cognitive behavioral therapy, motivational enhancement therapy, group counseling, and possibly pharmacotherapy.^14,15,16,17^

Practice facilitation is an approach to help practices improve quality of care through practice facilitators, trained personnel who collaborate with practices to build capacity for continuous and tailored improvement.^18^ Studies have shown practice facilitation can integrate evidence-based guidelines into practice, improve care delivery, and add new support services.^19,20,21,22,23^ A prior practice facilitation study with integrated electronic health record (EHR) support in one integrated health system increased screening and brief intervention for UAU but not more intensive treatment.^7^ Another multisite trial on practice facilitation combined with residency training efforts for UAU showed similar results.^11^ This trial sought to see if practice facilitation would improve recommended screening, brief primary care intervention, and treatment for UAU for a broad and representative group of primary care practices.

## Methods

### Overview

As part of the EvidenceNow Initiative, the Agency for Healthcare Research and Quality (AHRQ) funded 6 networks to study practice facilitation for UAU in 125 primary care practices.^24^ This article reports a primary care practice–level randomized clinical trial comparing practice facilitation to usual care for adults aged 18 to 79 years. Data were collected via medical record review and audio recording with transcription of practice facilitator meetings and exit interviews.

The study was approved by the Virginia Commonwealth University Institutional Review Board and follows the Consolidated Standards of Reporting Trials (CONSORT) reporting guidelines. The full protocol was previously published (Supplement 1).^25^ A waiver of informed consent was provided for the medical record review, and clinicians provided consent for the audio recordings. No changes were made to the methods, outcomes, or analysis after trial commencement.

### Setting

Primary care practices were recruited throughout Virginia between October 2019 and January 2023 with a goal of enrolling AHRQ’s requested 125 practices. We aimed to recruit a diverse sample of practices that represented the full scope of primary care and communities in Virginia. Recruitment strategies included flyers, emails, and personal connections facilitated by the Virginia Ambulatory Care Outcomes Research Network, multiple health systems, primary care specialty societies, Virginia’s Task Force on Primary Care, and telephone or email invitations.^26^

### Practice Block Randomization

Practices recruited within 3 weeks of one another were block randomized between immediate or delayed intervention in a 1:1 ratio. Blocks included multiple practices within the same health system or practice group. Odd number blocks included 1 additional intervention slot. Once enrollment for each block was complete, the practice facilitator provided the biostatisticians (R.S. and A.F.) a list of practices, and the biostatistician used the IML procedure in SAS statistical software, version 9.4 (SAS Institute), to randomize practices. Practices and facilitators were blinded to allocation to maintain allocation concealment.

Once randomized, practices and facilitators were not blinded to intervention group. Immediate intervention practices started practice facilitation once able to schedule meetings and provide EHR access for data collection. Delayed intervention practices received practice facilitation after a 6-month delay but provided EHR access at enrollment so data could serve as usual care (control) in the first 6 months. Due to administrative error, 4 practices in 1 block were inadvertently switched (2 practices randomized to delayed intervention received immediate intervention and vice versa; primary analysis based on intervention received).

### Patient Selection

From each practice, 60 patients aged 18 to 79 years who were seen the prior 3 months were randomly selected at baseline, 3 months, and 6 months of enrollment for medical record review. This approach ensured that clinicians were changing practice patterns for all patients and not a subset of potential study patients. Each period included a unique cohort of patients. Practices decided to screen at either wellness visits or any clinician visit. The practice facilitator abstracted a list of all eligible patients from the practice’s scheduling system, and the biostatisticians randomly selected 60 using the sample() function in R/RStudio, version 4.2.2 (Posit).

### Intervention

Intervention and control practices received the same intervention, with a 6-month delay for the control group. Practices were assigned a practice facilitator who led the practice facilitation. Facilitators were research team members with 3 to 10 years of practice facilitation experience (G.V., M.S.R., B.W.). Practices were asked to commit to an evidence-based process of screening, counseling, referral, and treatment for UAU. To do this, they formed a quality improvement team (1-4 clinicians, nurses, or office manager practice champions) who met with the facilitators 2 to 4 times to assess their practice’s current capacity, knowledge, and workflow, and create a practicewide change package, including standard workflow, documentation, screening, counseling, and treatment approaches.

All practice clinicians were asked to meet or asynchronously complete 3 educational sessions to learn about UAU, USPSTF screening recommendations, content of brief counseling interventions, motivational interviewing techniques, medications for AUD, and the practice workflow developed by practice champions. Practices were provided patient and clinician pamphlets, sample workflows, educational videos, pocket cards, assistance with EHR modifications, and an electronic library of community referrals. Baseline, 3-month, and 6-month screening, counseling, and treatment results were shared to monitor progress. The practice facilitator handbook (eAppendix 1 in Supplement 2) and all practice resources are available through an online tool kit.^27^

### Data Sources

Screening data were collected by medical record review.^28^ Structured data (eg, diagnoses, prescriptions, referrals, screening instrument) and text from office notes were reviewed. Demographics were as documented in the EHR. Any documentation or mention of alcohol use or screening, diagnosis, counseling, referrals, or prescriptions, including screening tool used, date documented, who documented, and where in the EHR it was documented, were recorded. Exit interviews were offered to all intervention practices, and 16 agreed to participate. Interviews asked about practice facilitation experience, changes made to screening and treatment, and effect on patient care. Practice facilitation meetings and exit interviews were audio recorded, transcribed, and thematically coded using a mix of a priori concepts derived from the Consolidated Framework for Implementation Research and an inductive immersion-crystallization approach to identify emergent themes.^29,30^

### Outcomes

Primary outcomes included whether patients were screened for UAU, instrument used, and whether patients screened positive. For patients who screened positive or had a diagnosis of risky drinking or AUD, primary outcomes also included whether they received brief counseling, referrals, or prescriptions.

Screening was defined as use of the AUDIT-C or SASQ. A positive screen was defined as an AUDIT-C score of 4 or higher for men 18 to 65 years old and 3 or higher for women 18 years and older or men older than 65 years, SASQ score of 1 or more episodes per year, or clinical diagnosis of AUD. Brief counseling was defined as any documentation of patient advice, provision of educational material, motivational interviewing, normative feedback, or follow-up visits for UAU. Referrals included individual or group therapies and structured and unstructured programs. Prescriptions included first-line medications (ie, naltrexone, acamprosate) and second-line medications (ie, disulfiram, topiramate, gabapentin) prescribed for AUD.

### Statistical Analysis

Each outcome was analyzed separately using SAS statistical software, version 9.4 (SAS Institute). Patient and practice characteristics were summarized overall and by treatment group. Outcomes were modeled using mixed-effect logistic regression models with a practice-level random effect. Fixed effects included group (2 levels), time (3 levels), and a group-by-time interaction while adjusting for both patient-level covariates (age, gender, race and ethnicity, and insurance type) and practice-level covariates (patient-centered medical home status, rurality, number of clinicians, and average patients seen per day). For each model, estimated percentages, 95% CIs, and *P* values for the difference in change from baseline to 6 months between groups were reported. Practice-level intraclass correlations (ICCs), the percentage of total model variability explained by the practice random effect, were calculated for each model.^31^ *P* values were multiplicity adjusted in each model using the Tukey honestly significant difference approach so that all were interpretable to a 5% significance level, and a Bonferroni correction to share significance across the 3 primary outcomes was applied so that each resulting *P* value was compared to .05/3 = .0167.

As a sensitivity analysis, we calculated outcomes as practices were randomized (intention to treat) for the 4 practices that unintentionally received the opposite intervention to which they were randomized. Accounting for variation due to clinician-based randomization and nesting of patients within clinicians,^32,33^ we calculated 90% power (with 5% type I error rate and intracluster correlation of 0.05) to detect a 10% difference in screening rates using AUDIT-C or SASQ (20% in control vs 30% in intervention) with 125 practices and 60 patient per practice assessment.

## Results

### Study Population

A total of 76 primary care practices enrolled. Nine dropped out after randomization but prior to data collection or intervention delivery, leaving 32 intervention and 35 control practices (Figure). Practices were broadly representative of primary care in Virginia, having different ownership models, representing 6 different health systems, and varying by specialty makeup, location, and experience with quality improvement (Table 1).^26^ Overall, clinicians attended 643 of 1072 (60.0%) practice facilitation meetings. Of the 76 practices, more elected to screen every visit vs wellness visits (38 [57%] vs 29 [43%]).

**Figure.**
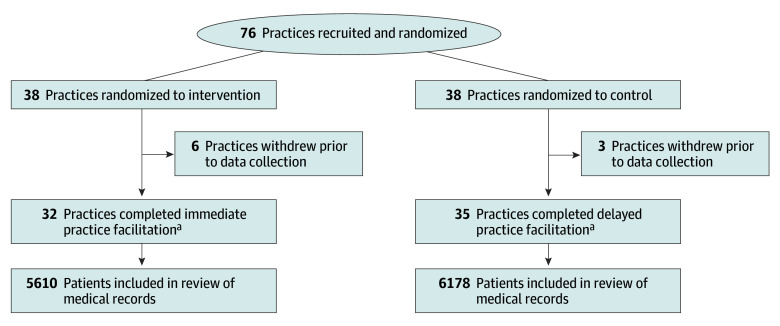
CONSORT Diagram Sixty patients from each practice at baseline, 3 months, and 6 months were included in the medical record review; however, the overall patient sample for the medical record review is slightly less than 60 patients for each practice at each time period because some practices did not have 60 patients in the 3-month prior period with a visit eligible for screening. ^a^Due to an administrative error, 2 practices randomized to immediate practice facilitation received delayed practice facilitation and vice versa. The 4 practices were in the same block and analyzed by intervention received.

**Table 1.** Participating Practice Characteristics

Characteristic	No. (%)			
	Total (N = 76)	Dropped out (n = 9)^a^	Intervention (n = 32)^b^	Control (n = 35)^b^
Practice type				
Family medicine	59 (78)	8 (89)	24 (75)	27 (77)
Internal medicine	2 (3)	1 (11)	0	1 (3)
Obstetrics and gynecology	4 (5)	0	3 (9)	1 (3)
Mixed primary/specialty	11 (15)	0	5 (16)	6 (17)
Practice location				
Urban	11 (15)	0	5 (16)	6 (17)
Suburban	24 (32)	1 (11)	11 (34)	12 (33)
Rural	41 (54)	8 (89)	16 (50)	17 (49)
Practice ownership				
University	1 (1)	0	1 (3)	0
Health system	54 (71)	6 (67)	23 (72)	25 (71)
Private	15 (20)	3 (33)	5 (16)	7 (20)
Community health center	6 (8)	0	3 (9)	3 (9)
Patient-centered medical home designation				
Yes	34 (45)	1 (11)	17 (53)	16 (46)
No	42 (45)	8 (89)	15 (47)	19 (54)
Part of accountable care organization				
Yes	47 (62)	6 (67)	20 (63)	21 (60)
No	29 (38)	3 (33)	12 (38)	14 (40)
Direct primary care practice				
Yes	2 (3)	1 (11)	0	1 (3)
No	74 (97)	8 (89)	32 (100)	34 (97)
Electronic health record				
Allscripts	1 (1)	0	1 (3)	0
Aprima	1 (1)	0	0	1 (3)
Greenway Intergy	1 (1)	0	1 (3)	0
NextGen	1 (1)	0	1 (3)	0
Practice Fusion	1 (1)	0	0	1 (3)
Capterra	1 (1)	0	1 (3)	0
Cerbo	1 (1)	0	0	1 (3)
eMDs	1 (1)	0	0	1 (3)
Athena	4 (5)	1 (11)	1 (3)	2 (6)
eClinicalWorks	8 (11)	0	5 (16)	3 (9)
Epic	54 (71)	6 (67)	22 (69)	26 (74)
MDVIP	1 (1)	1 (11)	0	0
Greenway Health	1 (1)	1 (11)	0	0
Screening instrument selected				
AUDIT-C	63 (94)^c^	NA	30 (94)	33 (94)
SASQ	4 (6)^c^	NA	2 (6)	2 (6)
Visit type selected				
Wellness visits	29 (43)^c^	NA	13 (41)	16 (46)
All visits	38 (57)^c^	NA	19 (59)	19 (54)
No. of clinicians participating, median (range)	5 (1-64)	NA	5 (1-64)	4 (1-51)
No. of patients seen per day per clinician, median (range)	20 (6-40)	NA	20 (15-25)	20 (6-40)

Abbreviations: AUDIT-C, Alcohol Use Disorders Identification Test-Consumption; NA, not applicable; SASQ, Single Alcohol Screening Question.

^a^ Practices dropped out after enrollment but prior to data collection or intervention delivery.

^b^ Reported by the intervention practices received.

^c^ Practices that dropped out were not included.

Overall, 11 789 patients were randomly selected for inclusion. The sample was representative of Virginia’s demographics, with 18.4% being Black, 7.8% being Hispanic, 23.2% having Medicare, and 13.4% having Medicaid (Table 2). Intervention and control groups were generally similar, although patients in the control practices were slightly more likely to be Black, non-Hispanic, older, and have Medicare or Medicaid, and control practices had a higher baseline rate of brief counseling for UAU (Table 2).

**Table 2.** Participating Patient Demographics^a^

Characteristic	Total (N = 11 789)	Intervention (n = 5610)	Control (n = 6179)
Race			
American Indian or Alaska Native	22 (0.2)	11 (0.2)	11 (0.2)
Asian	596 (5.1)	330 (5.9)	266 (4.3)
Black	2167 (18.4)	959 (17.1)	1208 (19.6)
Native Hawaiian or Pacific Islander	19 (0.2)	15 (0.3)	4 (0.1)
White	7550 (64.0)	3565 (63.6)	3985 (64.5)
>1 Race	77 (0.7)	39 (0.7)	38 (0.6)
Other^b^	250 (2.1)	124 (2.2)	136 (2.0)
Not reported	1108 (9.4)	567 (10.1)	541 (8.8)
Ethnicity			
Hispanic/Latino	921 (7.8)	563 (10.0)	358 (5.8)
Non-Hispanic	9369 (79.5)	4629 (82.5)	4740 (76.7)
Not reported	1499 (12.7)	418 (7.5)	1081 (17.5)
Age, y			
18-39	3339 (28.3)	1815 (32.4)	1524 (24.7)
40-59	4593 (39.0)	2080 (37.1)	2513 (40.7)
60-75	3488 (29.6)	1524 (27.2)	1964 (31.8)
≥76	369 (3.1)	191 (3.4)	178 (2.9)
Gender			
Men, including transgender men	4578 (38.8)	2107 (37.6)	2471 (40.0)
Women, including transgender women	7206 (61.1)	3498 (62.4)	3708 (60.0)
Nonbinary, gender nonconforming	5 (0.0)	5 (0.1)	0
Insurance			
Commercial	6585 (55.9)	3389 (60.4)	3196 (51.7)
Medicare	2731 (23.2)	1115 (19.9)	1616 (26.2)
Medicaid	1576 (13.4)	643 (11.5)	933 (15.1)
Dual enrolled Medicaid/Medicare	48 (0.4)	19 (0.3)	29 (0.5)
Self-pay	720 (6.1)	385 (6.9)	335 (5.4)
Tricare	129 (1.1)	59 (1.1)	70 (1.1)

^a^ Includes the sum of the patients included at baseline, 3 months, and 6 months. Demographics are reported as documented in patients’ electronic health records.

^b^ Other race represents when the practice or patient recorded race as “other” in the electronic health record.

### Screening Outcomes

Intervention practices had a greater increase in any documentation of alcohol use, from 75.7% (95% CI, 65.0%-83.9%) at baseline to 83.2% (95% CI, 74.7%-89.3%) at 6 months, compared to control practices, which decreased from 75.9% (95% CI, 69.7%-83.8%) to 70.6% (95% CI, 59.3%-79.8%) (*P* < .001 for difference in differences; ICC, 0.336) (Table 3). Intervention practices also had a markedly greater increase in screening with the AUDIT-C or SASQ, from 2.1% (95% CI, 0.5%-8.4%) at baseline to 35.5% (95% CI, 11.5%-69.9%) at 6 months, compared to control practices, which increased from 0.4% (95% CI, 0.1%-1.8%) to 1.4% (95% CI, 0.3%-5.8%) (*P* < .001 for difference in differences; ICC, 0.781). Intervention practices had a greater increase in identification of UAU from 4.6% (95% CI, 3.2%-6.6%) at baseline to 7.0% (95% CI, 5.0%-9.8%) at 6 months compared to the control practices, which only increased from 4.5% (95% CI, 3.1%-6.4%) to 4.9% (95% CI, 3.4%-6.9%) (Table 3), although the difference between groups was not statistically significant (*P* = .06; ICC, 0.123).

**Table 3.** Screening, Counseling, and Treatment Outcomes for All Patients Aged 18-79 Years With a Visit Eligible for Screening

Outcome	Adjusted % (95% CI)			Intraclass correlation	*P* value^a^
	Baseline	3 mo	6 mo		
**Screening results for all patients in practices determined eligible (N = 11 789)**					
Documentation of alcohol use^b^					
Intervention	75.7 (65.0-83.9)	74.2 (63.1-82.8)	83.2 (74.7-89.3)	0.336	<.001
Control	75.9 (69.7-83.8)	70.4 (59.1-79.6)	70.6 (59.3-79.8)		
Screening with AUDIT-C or SASQ					
Intervention	2.1 (0.5-8.4)	4.1 (1.0-15.7)	35.5 (11.5-69.9)	0.781	<.001
Control	0.4 (0.1-1.8)	1.9 (0.4-7.7)	1.4 (0.3-5.8)		
UAU identified					
Intervention	4.6 (3.2-6.6)	5.8 (4.0-8.2)	7.0 (5.0-9.8)	0.123	.06
Control	4.5 (3.1-6.4)	4.5 (3.2-6.5)	4.9 (3.4-6.9)		
**Counseling and treatment results for all patients who screened positive or had a diagnosis of UAU or AUD (n = 895)**					
Brief office intervention					
Intervention	26.2 (14.2-45.8)	54.2 (35.1-72.1)	62.6 (43.6-78.3)	0.309	.008
Control	45.5 (28.0-64.1)	54.6 (36.2-71.8)	55.1 (36.5-72.3)		
Referral for counseling and treatment					
Intervention	1.2 (0.3-4.9)	3.7 (1.1-11.6)	4.1 (1.3-11.9)	0.290	.91
Control	1.7 (0.3-5.9)	3.1 (1.0-9.6)	6.6 (2.4-17.0)		
Medication for AUD					
Intervention	0.4 (0.0-4.9)	0.7 (0.1-5.6)	1.4 (0.2-7.8)	0.253	.63
Control	2.2 (0.4-10.4)	3.2 (0.7-13.5)	2.7 (0.6-11.8)		
Any intervention or treatment					
Intervention	27.2 (14.2-45.8)	55.9 (36.5-73.7)	64.0 (45.0-79.5)	0.313	.004
Control	46.7 (28.8-65.4)	54.4 (35.8-71.8)	54.2 (35.5-71.8)		

Abbreviations: AUD, alcohol use disorder; AUDIT-C, Alcohol Use Disorders Identification Test-Consumption; SASQ, Single Alcohol Screening Question; UAU, unhealthy alcohol use.

^a^ *P* values compare the difference in differences between 6 months and baseline for the intervention and control groups.

^b^ Any documentation of alcohol use, including nonstructured notations in text about alcohol use, use of recommended screening instruments, and diagnosis of UAU or AUD.

### Brief Intervention and Treatment Outcomes

For patients who screened positive, there was a marked increase in brief intervention for intervention practices, from 26.2% (95% CI, 14.2%-45.8%) at baseline to 62.6% (95% CI, 43.6%-78.3%) at 6 months, compared to control practices, which increased from 45.5% (95% CI, 28.0%-64.1%) to 55.1% (95% CI, 36.5%-72.3%) (*P* = .008 for difference in differences; ICC, 0.309) (Table 3). Referral for more intensive counseling and treatment had similar small increases from baseline to 6 months for both intervention, from 1.2% (95% CI, 0.3%-4.9%) to 4.1% (95% CI, 1.3%-11.9%), and control, from 1.7% (95% CI, 0.3%-5.9%) to 6.6% (95% CI, 2.4%-17.0%) (*P* = .91 for difference in differences; ICC, 0.290). Similar small increases occurred for medications for AUD from baseline to 6 months for intervention and control practices (Table 3).

Results were similar for practices that elected to screen at every visit vs wellness visits. In the intention-to-treat sensitivity analysis, intervention practices had similarly greater improvements than control practices, but benefits were expectedly attenuated (eAppendix 2 in Supplement 2).

### Qualitative Findings

During exit interviews, clinicians reported making substantial changes to how they screened and counseled for UAU (Table 4). Clinicians changed from asking nonspecific questions (eg, “Do you drink alcohol?”) to using recommended screening instruments. They reported more consistently identifying a wider spectrum of at-risk patients, specifically those with previously unidentified risky drinking. Clinicians also reported a marked increase in comfort and confidence counseling patients about risky drinking. Some clinicians had increased confidence offering medications, but more expressed concerns, largely due to skepticism of benefits and concern for adverse effects. Clinicians reported hesitancy to formally diagnose or document UAU due to concerns about how patients would perceive this information or the adverse consequences of labeling.

**Table 4.** Key Qualitative Themes From Practice Facilitation Sessions and Exit Interviews^a^

Themes and findings	Example quotes
Clinicians improved screening and counseling practices: Use of recommended screening tools helped identify patients at risk Increased alcohol screening supported other preventive care efforts Improved sensitivity to and counseling for lower-level risky drinking	“I think that was really positive…getting the validated tool added into our EMR, adding it into our [rooming] process, getting our nurses to help you do it. And I think it’s fed into our other social screening, preventative stuff.” “I did not [screen] the way I should, not with a validated tool, and not on a regular, consistent basis. But now…I’m doing it all the time.” “I feel like I will catch maybe a patient or 2 a week that they don’t have serious alcohol problems, but they do exceed some of the healthy recommended limits.” “I think it opened up some good discussions with our patients about what’s healthy alcohol use, or unhealthy, or high risk, without necessarily attaching a lot of judgment to it and putting it in more objective terms.”
Clinicians felt more competent and confident to treat UAU: Increased awareness of alcohol use risk factors and the benefits of treatment Increased confidence providing MAUD Improved culture of care around social and behavioral health issues	“I probably do screen a little bit more often. But more so I’m confidently offering treatment because of the study. And then definitely giving people information in hand. That’s something I was not doing before.” “No one in my practice, none of my faculty, including myself, and none of the residents felt comfortable doing MAUD before this study. And after the study, we were like, ‘It’s not that hard. We can all do it. It’s easy.’” “It was great because it pushed us back into the perspective of looking at social history and the importance of social determinants of health. That’s been a big change just in the culture of our practice of medicine.”
Clinicians were hesitant to diagnose and/or document UAU: Concerned about patient perception of UAU documentation Preferred to use vague or veiled language Lack of reimbursement for UAU-related activities led to poor documentation	“I do put it in the chart, but I try to not make [the diagnosis] because the notes are directly pushed to the patient.” “I don’t use terms like ‘alcoholism’ or ‘abuse’ or anything like that. I just do the ‘alcohol misuse.’ I think patients are more accepting of that. My documentation is very general, like, ‘This is what healthy alcohol use looks like.’” “We may not specifically code or bill for it [UAU diagnosis or counseling] because they’re never going to pay for it anyway.”
Clinicians expressed concern about using MAUD: Concerned that medications may not help Concerned about medication adverse effect profile Biased by adverse effects of older medications (eg, disulfiram)	“I know of a few residents I’ve worked with who do MAUD with their patients, and it just hasn’t been successful. They start, and the patient relapses or just decides it’s not working and doesn’t take it anymore.” “Especially if it’s a patient I haven’t worked with, a new patient or something, I question if we are going to do more harm than help.” “Back in the day when I learned about [MAUD] the side effect profiles were pretty nasty. It’s kind of harsh…people get pretty sick if they take the medicine. So we don’t really use much of that anymore.”

Abbreviations: EMR, electronic medical record; MAUD, medications for alcohol use disorder; UAU, unhealthy alcohol use.

^a^ Derived from interviews with clinicians and practice leaders at 16 of 32 intervention practices.

## Discussion

Practice facilitation was a highly effective intervention to improve screening and counseling for UAU in everyday primary care practices, but it only marginally increased detection of UAU and did not increase more intensive treatments like referrals or medications. Yet, given that the evidence reviewed by the USPSTF showed that the primary benefit of UAU screening was from brief interventions to reduce risky drinking,^13^ practice facilitation likely had a considerable population benefit.

Consistent with other studies, we found that screening and brief intervention for UAU are poorly delivered in primary care^9,10,11,12^ and that practice facilitation can help clinicians to implement more evidence-based practices.^7,11^ Transcripts of practice facilitation sessions showed that key elements included helping clinicians understand the recommended screening instruments, feel more confident and competent with brief interventions, adapt and redefine workflows, designate staff tasks, make EHR changes, review performance feedback, and learn from others’ experiences. Practice champions were an essential change component, consistent with prior literature.^34,35^ Interestingly, in many health systems and practice groups, intervention practices implemented changes that affected all practices, such as adding the AUDIT-C to the EHR.^28^ While these interventions could have benefitted control practices, similar changes in screening and counseling were not observed, reinforcing the need for practice facilitation to implement changes.

Baseline data showed that clinicians asked patients about and documented alcohol use, though not with recommended screening instruments. This resulted in missing most cases of UAU and was an inefficient and ineffective use of valuable time. Many clinicians reported knowing their patients well and were surprised when patients screened positive using a screening instrument. While there was a trend toward greater identification of UAU with practice facilitation, it did not reach statistical significance, and the 7.0% detection rate fell short of the known 21% rate of UAU in the general population.^6^ Possibly with more time, we would observe clinicians detecting more cases of UAU. Other studies have shown that patient self-report using the AUDIT-C results in much higher detection rates than clinician administration (14.7%-36.6% of people screened vs 1.6%).^36^ Future efforts could focus on automating the EHR to send patients screeners prior to visits.

The scope and reach of this simple intervention outpace most preventive service implementation studies.^37,38,39^ Collectively, the practices that completed practice facilitation saw 412 409 unique adults in 2021. The observed 33.4 percentage point increase in screening with recommended screening instruments compared to control means that 114 604 additional patients could be screened annually. The observed 2.4 percentage point increase in UAU identification means that an additional 8235 patients could be identified with UAU and 5155 could receive brief interventions. This could be further increased with improvements to increase UAU detection.

In this study, like others,^7,9,10,11,12^ only 0.4% to 6.6% of patients with UAU received more intensive interventions, such as referral or medications. Referrals were primarily made for patients with AUD, which is less common than UAU. In practice interviews, clinicians reported both a limited number of programs to refer patients and low interest from patients.^40^ Clinicians also reported a strong hesitancy to prescribe medications even after learning about their safety and efficacy. Consistent with other studies, some clinicians viewed prescribing medications as complex and time-consuming, often believing patients may not be good candidates or nonadherent, or even having negative personal beliefs about medications.^41^ Yet, these medications are simple, safe, effective, and within the scope of primary care.^42^

### Limitations

This study has several limitations. First, we only recruited 76 practices, not AHRQ’s requested 125 practices. This largely occurred because the start of recruitment coincided with the start of the COVID-19 pandemic. Second, 4 practices in 1 randomization block were placed in the incorrect group due to administrative error. Since all practices received the reverse of randomized, and this stage of the trial was not blinded, this error unlikely introduced a systematic bias. Sensitivity analysis showed that intervention practices received greater benefit than control practices, but benefits were attenuated. Third, we relied on medical record review to document brief counseling. Clinicians generally did not document details about counseling, beyond that it occurred, and we were unable to assess the elements and quality of counseling. Finally, the rate of brief counseling was initially higher in the control group. Practices were grouped by health system, practice group, and region for randomization to limit this risk. Baseline data were not available until several months after randomization, so it was not a failure of allocation concealment. It is unclear why this discrepancy existed beyond random chance.

## Conclusions

Results of this cluster randomized clinical trial show that more work is needed to improve screening, brief intervention, and treatment of UAU in primary care. While screening dramatically increased at 6 months, intervention practices likely only identified a third of people with UAU. Brief interventions by primary care clinicians could also be improved. Clinicians were provided educational pamphlets to give patients and taught to encourage patients to complete drinking diaries and have follow-up counseling visits. These rarely occurred. Furthermore, clinicians tended to rely on more traditional group counseling programs for referral. Effective individual treatments, like cognitive behavior therapy, were rarely used. These referral resources may be more accessible and acceptable to patients. Finally, clinicians need more systems’ support for screening, counseling, referral, and treatment. As an example, while EHRs can field and document screening instruments like the AUDIT-C or prescriptions, no practices in this study were able to integrate into their EHRs more robust, automated previsit dissemination of screening questionnaires, counseling support, educational material, community referrals, or coordination of care across interdisciplinary care team members.^28^

Screening and counseling for UAU is feasible in primary care, and this pragmatic adaptive approach to implementing tailored practice facilitation dramatically improved care. Similar support should be extended more broadly to primary care nationally, and future practice facilitation studies should focus on improving detection, brief intervention, and treatment of UAU.

## Notes

Supplement 1. Trial Protocol

Supplement 2. **eAppendix.** Practice Facilitator Guide **eAppendix 2.** Sensitivity Analysis – Screening, Counseling, and Treatment Outcomes for All Patients Aged 18 to 79 Years with a Visit Eligible for Screening

Supplement 3. Data Sharing Statement
