# Supplementary material for: Practice Facilitation to Address Unhealthy Alcohol Use in Primary Care: A Cluster Randomized Clinical Trial
Source: JAMA Health Forum. 2024 Aug 9;5(8):e242371. doi: 10.1001/jamahealthforum.2024.2371 (PMC11316228; doi:10.1001/jamahealthforum.2024.2371)
Supplement: Supplement 1. — Trial Protocol [file jamahealthforum-e242371-s001.pdf]

Practice Facilitation to Promote Evidence-based Screening and Management of Unhealthy Alcohol Use in Primary Care: Study Protocol

PROJECT SUMMARY

Unhealthy alcohol use is the third leading cause of preventable death in the US. Evidence shows that screening for unhealthy alcohol use and providing persons engaged in risky drinking with brief behavioral counseling interventions improves health outcomes, collectively termed screening and brief intervention (SBI). For moderate or severe alcohol use disorder (AUD), medications for opioid use disorder (MAUD) is effective. Despite clear evidence of effectiveness, only 13% of primary care patients are screened with a standard instrument and only 6.7% of adults with AUD receive treatment. We believe that underutilization of SBI and MAT are driven by both a misunderstanding of the role and effectiveness of primary care in addressing unhealthy alcohol and limited practice resource and infrastructure. To promote the dissemination and implementation of evidence-based strategies to address unhealthy alcohol use throughout Virginia, we have extended our EvidenceNow collaboration to include addiction medicine experts at Virginia Commonwealth University, the Virginia Ambulatory Care Outcomes Research Network (ACORN), our state's family medicine residency training programs, and our state's Community Service Boards. We propose a practice-level cluster randomized trial with wait list control. 125 primary care practices in five regions throughout the state, each centered around a residency site for educational support, will receive a practice facilitation intervention to implement screening, counseling, and treatment for unhealthy alcohol at intervention start or 6-month delay. Guided by the identified EvidenceNow key drivers for change, practice support will include practice facilitation, education and training, shared learning and best practices, screening and counseling toolkits, data support, and assessment with feedback. Each practice will identify a clinician, nurse, and administrator champion to locally lead efforts and participate in learning collaboratives. Practices will design and implement screening, counseling, and treatment processes and operational changes, adapting their implementation strategy based on experiences and findings from other sites. We will conduct a mixed methods analysis. Primary outcomes will include the increase in screening for unhealthy alcohol use, increase in provision of brief counseling interventions and MAT, and reduction in alcohol intake for patients after practices receive practice facilitation. We will use the consolidated framework for implementation research to code and rate practice facilitation (e.g. dose, mode, reach) and practice implementation strategies (e.g. SBI and MAT strategies and tools implemented) on outcomes. Data sources will include practice facilitator field notes and interviews, chart reviews, patient survey, clinician survey, All Payer Claims Data, and qualitative interviews. We will administer the patient survey at baseline, 3 months, and 6 months after the intervention. Among patients aged 18 to 75 with an office visit the prior month, we will randomly select 60 to survey. In addition to our internal evaluation, we will participate in the external collaborative evaluation and dissemination activities with AHRQ throughout the project.

SPECIFIC AIMS

Screening and counseling for unhealthy alcohol use is one of the most poorly delivered health behavior counseling services in primary care. We believe that this is because most primary care clinicians do not understand that they can improve health outcomes by identifying risky drinking and providing brief counseling. Instead, most clinicians focus on identifying and treating moderate to severe AUD, which is more difficult to manage, requires significantly more patient support, and benefits from MAT, which primary care clinicians are uncomfortable prescribing. Adding to this, most existing practice infrastructure (electronic health record alerts and reminders, screening instruments) and resources (patient educational and self-management resources, referral network) have not been developed to support screening and counseling interventions for risky drinking. We propose to provide practice facilitation to guide practices in developing a shared understanding of the evidence and standardized protocols for screening, counseling, and treatment of risky drinking and AUD. As part of this dissemination and implementation effort, the intervention will be adaptive, and we will evaluate three metrics utilizing a cluster randomized, wait list control design methodology. **By the second year of the study, all practices (intervention and control) will receive the intervention – practice support to implement screening, counseling, and treatment of unhealthy alcohol.** We will compare changes from baseline to 3- and 6-months post-intervention for intervention versus control practices. The baseline to 3-month comparison will assess effectiveness, while the baseline to 6-month comparison will assess maintenance.

<u>Aim 1 (Screening):</u> To evaluate whether practice facilitation increases screening rates for unhealthy alcohol use in primary care. From patient postal survey data, chart reviews, and All Payer Claims Data (APCD), we will determine whether there is a greater increase in screening for unhealthy alcohol use at 3 and 6 months for patients in intervention practices versus wait list control practices.

Hypothesis #1: Compared to control practices, 10% more patients in intervention practices will report being asked about alcohol use (increase from 78% to 86%) and 50% more patients in intervention practices than control practices will have a documented screen using AUDIT-C or SASQ (increase from about 20% to 30%).

<u>Aim 2 (Treatment):</u> To evaluate whether practice facilitation increases treatment for unhealthy alcohol use in primary care. From patient postal survey data, chart reviews, and APCD, we will determine:

- <u>Sub-aim 2a</u> Whether there is a greater increase in counseling patients with risky drinking (more than 14 drinks per week for men, 7 drinks per week for women, or more than 3 drinks per occasion) at 3 and 6 months for intervention versus wait list control practices;
- <u>Sub-aim 2b</u> Whether there is a greater increase in MAT for patients with moderate to severe AUD at 3 and 6 months for intervention versus wait list control practices.
- <u>Sub-aim 3b</u> Whether patients who report risky drinking reduce the amount they drink in 6 months.

Hypothesis #2: Compared to control practices, 50% more patients in intervention practices with risky drinking will report or have documented treatment (brief counseling and/or MAT) (increase from 20% to 30%).

<u>Aim 3 (Practice Implementation and Support Moderators):</u> To understand the practice implementation strategies and practice support factors that influence the effectiveness of the intervention in promoting routine screening for unhealthy alcohol use. From practice facilitator field notes, facilitator interviews, and the clinician survey, we will code and qualitatively rate consolidated framework for implementation research (CFIR) constructs that influence intervention implementation effectiveness.

- <u>Sub-aim 3a</u> What practice implementations strategies most benefit a practice's ability to implement screening, counseling and treatment protocols to address unhealthy alcohol use.
- <u>Sub-aim 3b</u> What practice facilitation factors influence implementation success.
- <u>Sub-aim 3c</u> How community, organization and practice-level factors impact implementation efforts.
- <u>Sub-aim 3d</u> How practices adapt implementation strategies to reflect local needs and challenges.

Innovation and Impact: Using an established EvidenceNow partnership, this study will systematically disseminate and implement evidence-based screening, counseling, and treatment recommendations for unhealthy alcohol use through a practice facilitation intervention. Given the low uptake of this preventive service and adverse consequences of unhealthy alcohol use, this study has great potential to improve health in Virginia. Additionally, the systematic implementation evaluation can inform future practice support efforts for other evidence-based services and for other settings.

APPROACH

Project Overview

We propose a cluster randomized trial with wait list control (Figure 3). We will recruit 125 primary care practices distributed across five regions of the state. Each region is centered around a local residency site that will serve as an educational hub to help with practice recruitment and support. All practices will receive the intervention by the end of the second year. To facilitate feasibility, we will sequentially initiate the intervention in each region. Practices will be randomly allocated in a 1:1 ratio to receive practice facilitation at startup or after 6 months delay. Practice facilitation will include provision of a facilitator, education and training, shared learning and best practices, screening and counseling toolkits, data support, and assessment with feedback. Practice activities will be locally led by a self-selected clinician, nurse, and administrator champion. We anticipate that the intervention will adapt and evolve over the regional rollout. Using mixed methods, we will assess the increase in screening for unhealthy alcohol use, increase in provision of brief counseling interventions and MAT, reduction in alcohol intake, and influence of practice facilitation (e.g. dose, mode, reach) and practice implementation strategies (e.g. SBI and MAT strategies and tools implemented and how implemented) on outcomes. Throughout, we will participate in the external collaborative evaluation and dissemination activities with AHRQ throughout the project.

Figure 3. Consort – Implementation Study Flow Diagram

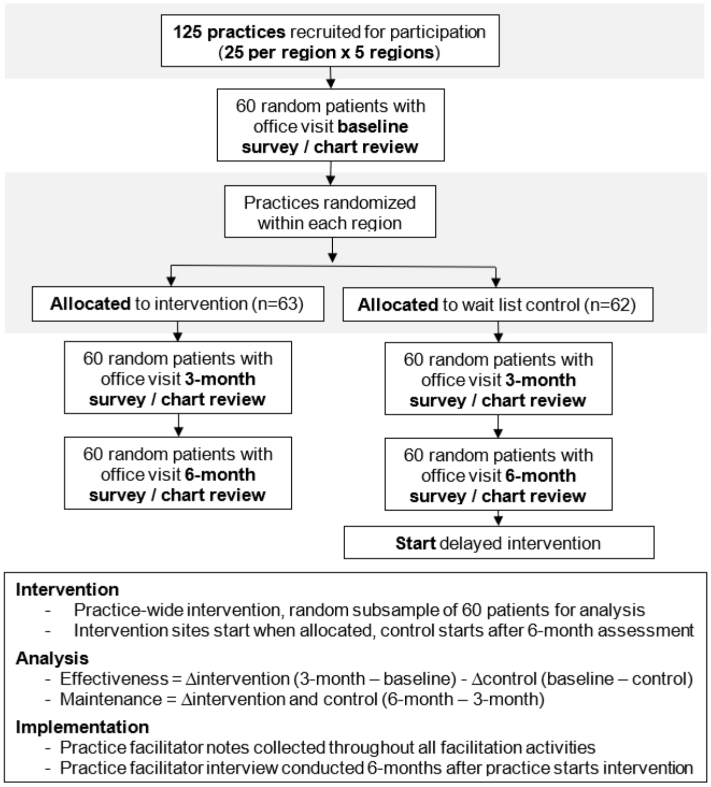

Section 1: Project Team and Community Partnerships

This project will build on our successful EvidenceNow study, "Restoring Primary Care in Virginia: PCOR Learning as a Pathway to Value." In EvidenceNow, <u>we created a highly effective partnership called *Heart of Virginia Healthcare*</u> (Figure 4). We recruited 249 small to medium sized primary care practices, delivered a practice facilitation-based intervention to promote the ABCS of cardiovascular prevention, and demonstrated practice improvements in delivery of both aspirin and statin chemoprevention. To ensure the success of this proposal, <u>we will expand our partnerships to further include Virginia's statewide family medicine residency training programs, the ACORN practice-based research network, addiction medicine specialists from the VCU's Alcohol Research Center, Virginia's network of CSBs, and VCU's Department of Biostatistics</u>.

Figure 4. Our Successful EvidenceNow Partnership (2014-2018)

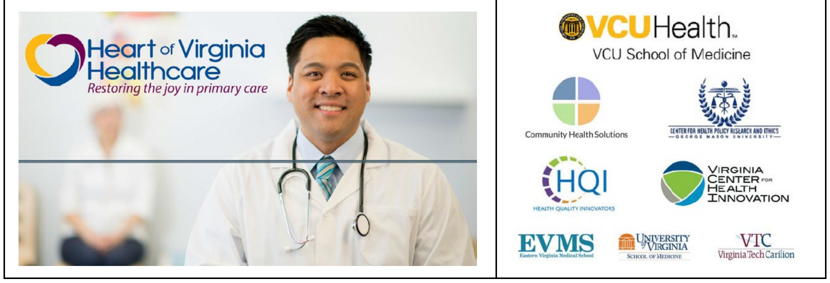

To conduct this study, we have six key groups that will work together – project leadership, practice facilitators, regional-residency hub leads, practice leads, advisors, and partner organizations. Organizationally, the project leadership and practice facilitators will meet in person every two weeks to plan, review, and coordinate day to day study operations. This will allow leadership to monitor and support all practice facilitation activities throughout. The regional-residency hubs will be asked to join these meetings during planning and fielding periods in their region. Quarterly, all groups (except practice leads) will meet in person and virtually for overall project updates, strategic planning, and to coordinate dissemination activities. During their study period, each region will have quarterly virtual learning collaboratives to share lessons learned (see below for details). Project leadership, practice facilitators, the regional-residency hub lead, and the practices' leads will attend.

Project Leadership

VCU will serve as the parent organization for this proposal in collaboration with our partner organizations. As described above, VCU has a track record of successfully supporting practice transformation, is a leader in promoting evidence-based guidelines, and has developed much of the infrastructure needed to support practice facilitation for unhealthy alcohol screening and counseling. This partnership will be guided by a leadership committee. The leadership committee will meet every two weeks throughout the project to plan and coordinate activities and monitor progress. Members include:

- Principal Investigator: Alex Krist MD MPH – Director of Research, Department of Family Medicine and Population Health, VCU; Director ACORN; Vice Chair USPSTF.
- Implementation Co-Leads:
  - Beth Bortz MPP – President & CEO, Virginia Center for Health Innovation.
  - Anton Kuzel MD MHPE – Chair, Department of Family Medicine and Population Health, VCU; Principal Investigator for EvidenceNow.
  - John Epling MD MSEd – Director of Research, Family and Community Medicine, VirginiaTech Carilion; Medical Director of Employee Health and Wellness, Carilion Clinic; member USPSTF.
- Practice Support/Training Design Co-Leads:
  - Dace Svikas PhD – Professor, Departments of Psychology, Psychiatry and Obstetrics/Gynecology, VCU; Director AWHARE Program (Addiction and Women's Health: Advancing Research and Evaluation); Motivational Interviewing Network of Trainers certified.
  - Albert Arias, MD MS – Associate Professor, Department of Psychiatry; Investigator and educator for AUD and MAT.
- Outcomes Evaluation Lead: Roy Sabo PhD – Associate Professor, Department of Biostatistics, VCU.
- Implementation Evaluation Lead: Marshall Brooks PhD – Cultural Anthropologist, VCU.

Practice Facilitators

We will have three full time practice facilitators to support the practices. The practice facilitators will be part of the VCU research team and integrated into all team activities. The facilitators have ten-, three-, and three-years' experience with research and practice facilitation, respectively. Facilitator training includes reading and understanding the EvidenceNow Tools for Change for Practice Facilitators, co-creating the practice change package to guide activities based on the EvidenceNow toolkit, observing a third of each others' facilitation activities with assessment and feedback by the co-facilitator, and possible formal training at workshops during the annual International Practice Facilitator Conference.

Regional-Residency Hub-Leads

There will be a lead for each of the five regional-residency hubs. Leads live and practice in their region, are faculty within the local residency, know or have trained many of the community clinicians, and are considered thought leaders. Regional-residency leads will work closely with the leadership committee and the practice facilitators throughout the study. Specific activities will include recruiting at least 30 practices (anticipating 25 will participate), providing in collaboration with other local residency faculty academic detailing for each practice, being available for support and expert consultations, encouraging and monitoring data collection, and participating in outcomes and implementation analysis.

- Richmond lead: Victor Agebeibor MD; residency hub – Saint Francis Family Medicine Residency.
- Fairfax lead: Marc Childress MD; residency hub – Fairfax Family Medicine Residency.
- Roanoke lead: John Epling Md MSEd; residency hub – VirginiaTech Carilion Family Medicine Residency.
- Front Royal lead: Jonathon Winters MD ; residency hub – Shenandoah Valley Family Medicine Residency.
- Newport News lead: Bradley Touchet MD; residency hub – Riverside Family Medicine Residency.

Practice Leads

Each participating practice will be asked to identify a clinician, nurse, and administrator champion to participate. These champions will meet regularly with the practice facilitator to design and implement screening, counseling, and treatment protocols for their practice; and to adapt their implementation strategy based on their experiences and findings as well as from other sites.

Advisors

Three advisors will provide the leadership committee expert consultations. Specific input will include finalizing the design of the study, refining and adapting the intervention, data collection, outcomes and implementation analysis, and dissemination of findings. These advisors are leaders in their field and our team

has worked with them on a range of projects for the past decade or more. They include Michael Parchman (practice facilitator advisor), Perry Dickinson (implementation advisor), Deborah Cohen (evaluation advisor).

Partner Organizations

Our partner organizations represent key stakeholders in Virginia involved with practice transformation and quality improvement, clinician education, healthcare delivery, and addiction medicine service delivery (Table 5). VCHI, VCU, and ACORN have longstanding relationships with all these organizations and have collaborated on similar projects in the past. Throughout the study, leadership committee members, regional-residency hub leads, and practice facilitators will work with partner organization members to support study activities. See attached letters of support.

Table 5. Partner Organizations

Practice transformation partners	• VCHI<br>• Virginia Academy of Family Physicians (VAFP)<br>• Virginia Department of Health (VDH) (Norm Oliver)<br>• Dept of Behavioral Health & Developmental Services (DBHDS) (Hughes Melton)<br>• Dept of Medical Assistance Services (DMAS) (Kate Neuhausen)<br>• American College of Physicians (ACP), Virginia Chapter<br>• Wright Center (VCU Center for Clinical and Translational Research)	
Clinician education	• VCU Department of Family Medicine and Population Health<br>• VCU family medicine residency programs<br>• VirginiaTech family medicine residency programs	
Primary healthcare delivery	• VCU Health System<br>• Bon Secours<br>• Valley Health<br>• Privia Medical Group	• Carilion Health System<br>• Inova Health System<br>• Riverside Health System<br>• Sentara Healthcare
Addiction medicine service partners	• Virginia Association of Community Service Boards<br>• NIDA Clinical Trials Network<br>• AWHARE Program (Addiction and Women's Health: Advancing Research and Evaluation)	

Section 2: Access to and Recruitment of Primary Care Practices

Proposed Region – Virginia

We propose to conduct this study from five regional hubs that span and include the entire state of Virginia. This is an exciting time to do quality improvement research in Virginia. Beginning January 2019, Virginia will be the first state to expand Medicaid in the post-2016 election era. As a result, more Virginians will have access to care. Statewide investments are being made by local and state government and healthcare systems to be able to accommodate the estimated 400,000 Virginians that will gain health insurance as a result. Additionally, state leadership, under the direction of family physician leaders (e.g. Norm Oliver, Kate Neuhausen, and Hughes Melton) are promoting programs to address integrated primary care and behavioral health and addiction medicine services. These programs are showing success for reducing opioid addiction. Knowledge from these experiences can be applied to unhealthy alcohol use. Furthermore, most practices in the state are participating in value-based payments. Given the strong influence of health behaviors on health and wellbeing, more effective screening and counseling for unhealthy alcohol can improve the quality of care and reduce costs, aligning with practice financial interests.

Figure 5. Binge Drinking in VA

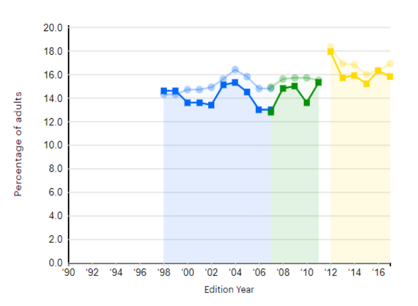

Virginia is home to more than 8.1 million people residing in 134 cities and counties spread across 40,327 square miles. In Virginia, 15.8% report binge drinking in the past month (Figure 5). Rates are higher among males (male vs. female: 20.2% vs. 11.8%), younger adults (18-44 vs. 45-64 vs. 65+ years: 24.4vs. 12.4% vs. 3.3%), more educated (less than high school vs. college grad: 8.9% vs. 15.7%), higher income (<$25,000 vs. $75,000+: 10.0% vs. 17.9%); and it is equally common among all races/ethnicities (13.0%-17.0%) and in urban, suburban, and rural communities (9.3%-10.6%). Similar trends are seen in Virginia with drinking more than recommended with an overall rate of 17.4% and a rate of 10.2% to 24.2% depending on the subpopulation (10.2% for less than high school education and 24.2% for age 18-44 years). Based on AHRQ's National Health Quality Report, Virginians do report good access to care (pre-Medicaid but only 42.9% of adults in Virginia treated for a substance use disorder complete treatment.

Healthcare in Virginia

Based on our work to assess primary cares capacity to handle Medicaid expansion, we have identified in Virginia: 1934 family medicine physicians; 1439 internal medicine physicians; 42 medicine-pediatric physicians; 17 preventive medicine physicians; 2182 family, adult, and geriatric nurse practitioners; and 694 physicians assistants working in family, internal, and geriatric medicine. Analysis of these clinicians by address suggests that there are 1836 independent primary care practices and 140 FQHC sites. Most of these practices qualify

for participating in this study; 249 of these practices participated in EvidenceNow; 155 are active members of ACORN; and all are being surveyed as part of our Medicaid expansion assessment.

Practice Recruitment

We will recruit 150 small to medium sized practices from 5 regions throughout the state for participation in this study and anticipate 25 will drop out (n=125 receiving practice facilitation). Regions will be centered around each of the 5 VCU and VirginiaTech Carilion family medicine residency training centers. <u>Our recruitment strategy is based on relationships and connections.</u> Each regional hub will be charged with recruiting 30 local practices. We will use five techniques to approach practices for recruitment (presented in order): (1) residency faculty will draw on local connections and graduates, (2) the ACORN practice advocate will approach local ACORN practices, (3) Kuzel and Bortz will approach prior EvidenceNow participants, (4) VCHI staff will approach prior VCHI practice and health system partners, and (5) VCU family medicine staff will approach practices indicating an interest in research participation on our Medicaid expansion primary care survey not included in the other groups.

**Table 6. Value Proposition for Study Participation**	
Care improvements	• Improved screening and counseling for unhealthy alcohol use
Practice improvements	• Provision of a practice facilitator<br>• Access to practice tools for screening and counseling<br>• Shared learning from other practices<br>• Benchmarked screening and counseling rates
Clinician improvements	• Improved satisfaction being better able to care for patients<br>• Part IV maintenance of certification (MOC) credit<br>• Continued medical education (CME) credit
Financial improvements	• Potential performance bonuses<br>• Screening and counseling billing
Alignment with other initiatives	• Statewide effort to address substance misuse, mental health<br>• Efforts to expand access to care

Our recruitment and engagement strategy will be based on our deep understanding of the challenges facing primary care practices in today's uncertain environment. We anticipate that the greatest barrier to participation will be feeling too busy to add one more thing. To address this barrier, we will provide clear and candid expectations of the level of effort required for participating practices, including responsibilities for data collection and reporting. Our recruitment message will include a strong value proposition in which the anticipated costs of participating will be more than justified by the expected benefits. The value proposition is shown in Table 6, above. Additionally, our study is purposely designed such that the research components (e.g., chart reviews and patient surveys) will have low impact on practices and be feasible (e.g., only requiring simple EHR queries of patient names, addresses, and appointments).

We believe that this recruitment approach will be successful. In 2003, VCU was the fifth university in the nation to train 1000 family medicine physicians. More than 60% of graduates remain in the state, creating a wide network of potential participants. The ACORN network has been successfully conducting research since 1996 with no practices withdrawing from the network and many participating in research for over two decades. Figure 6 shows the regions, residency hubs, and ACORN practice members. We plan to recruit 150 practices for participation, anticipating a 15% dropout rate, similar to our EvidenceNow study. To recruit 150 practices, we need 97% of ACORN practices (n=155) or 60% of EvidenceNow practices (n=249) or 8% of all Virginia primary care practices (n=1976) to agree to participate. Historically, we have had a 60% study recruitment rate when approaching ACORN practices and a 30% recruitment rate when approaching research naïve practices. These rates will be enhanced using our residency connections for recruitment. <u>Overall, we are assuming that at least 50% of practices we approach will participate in this study</u>. We plan to recruit a diverse range of practices for participation with respect to ownership, infrastructure, and resources as well as in terms of communities served and assume participating practices will have similar characteristics to our ACORN membership (Table 7).

Figure 6. ACORN Practices and Family Medicine Residency Training Centers

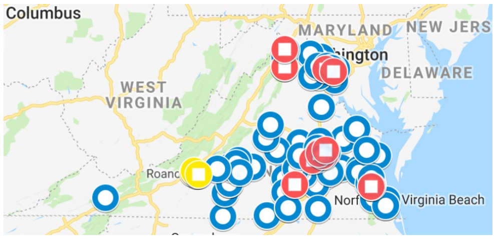

Blue – ACORN practices; Red – VCU family medicine residencies; Yellow – VirginiaTech Carilion residency

We are not aware of any data on the current rates of unhealthy alcohol use screening in primary care in Virginia, but suspect it is no better than reported national averages. Our baseline patient survey and chart review will establish current practice in Virginia, which is sorely needed data. We know that the uptake of integrated behavioral health is very low in Virginia, with few practices (<15%) reporting on our Medicaid survey co-location of behavioral health providers, partner organizations to handoff or refer patients, routine information exchange with behavioral health providers, or personal communications with behavioral health providers. In

fact, the Farley Center is conducting a policy analysis on behavioral health as part of our state's preparation for Medicaid expansion and they have identified that only a third of behavioral health providers in Virginia even take insurances. Many communities do have health system-based programs to assist individuals with alcohol misuse. However, these programs are often designed to treat patients with more severe AUD, not to provide counseling for risky drinking as recommended by the USPSTF. Also, every county and city in Virginia has an assigned CSB, which is the primary point of entry into the Commonwealth's public behavioral health and developmental services system. CSBs provide treatment for mental health issues that contribute to unhealthy alcohol use and substance use and addiction. Unfortunately, most primary care practices are not aware of the services their CSB providers nor do they have established working relationships. This limited infrastructure and support means that primary care needs to take a lead on screening and counseling for risky drinking and primary care needs support to establish connections with local programs to help patients with moderate to severe AUD, as we propose in this study.

**Table 7. Anticipated Characteristics of Study Practices (ACORN Profile)**	
Number of health systems	9
Number clinicians	
Average	5.2
Range	1 to 54*
Location	
Urban	30%
Suburban	55%
Rural	15%
Other	
Residency	4%
FQHC	5%
Safety net	10%
* Practices with >10 clinician FTEs will be excluded	

To ensure study feasibility, we will have a staged roll out for each region, with each regions' start separated by 3 months (see Project Timeline, below). In each region, we will spend 3 to 14 months recruiting practices, 2 months conducting the baseline patient survey and chart review, and then randomize practices to start the intervention. Practices recruited within three weeks of one another will be block randomized between immediate intervention or delayed intervention in an approximate 1:1 ratio. Blocks will generally include multiple practices within the same health system or practice group. Blocks with an odd number of practices will have one additional intervention slot. Blocks with only one practice will be randomized between groups with equal probability. We anticipate that less than 15% of practices will drop out and if they do, it will be during the baseline survey and chart review. Any practice that cannot complete these baseline assessments will be replaced. Research personnel will assist any practice that completes the baseline survey or chart review but cannot do the 3- or 6-month survey.

Section 3: Approach to PCOR Dissemination and Implementation

Intervention Overview

To disseminate and implement screening, counseling, and treatment of unhealthy alcohol, we are proposing to provide practice support for practices in making operational and process changes. The combination of support and practice changes are designed to address the six key drivers identified through EvidenceNow that primary care practices need to make to build their capacity to implement the best evidence. How these drivers are addressed are shown Table 8. Based on the PHARIS Diagnostic Grid and evidence about why primary care practices do not provide more screening, counseling, and treatment for unhealthy alcohol use, our intervention has an emphasis on ensuring clinicians understand the preventive service and infrastructure support.

TABLE 8. Overview of EvidenceNow Key Change Drivers and Strategies to Address Drivers

Key Driver	Change Strategy	How Provided
Seek and implement evidence	Develop a process to search for new evidence	- Educational sessions to share evidence<br>- Research team track and disseminate new evidence
Implement quality improvement	Develop an interprofessional quality improvement team	- Form practice quality improvement team<br>- Practice facilitator provide support
Optimize health information systems	Develop standard documentation; determine if EHR supports measuring care	- Toolkit for EHR support<br>- APCD to supplement monitoring implementation<br>- Shared learning on EHR use for documenting and monitoring care
Create care teams	Establish care teams and delineate roles	- Practice team to define screening, counseling, and treatment processes and care team roles
Engage with patients and families	Identify patients effected by the evidence	- Define process to screen patients<br>- Employ motivational interviewing for counseling
Nurture leadership	Forge a vision for adapting new evidence	- Learning collaboratives to learn from other practices<br>- Research team to disseminate identified best practices

Identifying PCOR findings

As an adaptive intervention, we anticipate that the approaches and resources practices need will evolve over the course of the study. Accordingly, the research team will systematically identify and disseminate both new and established PCOR findings in the context of practical lessons from preceding regions. PCOR findings will be disseminated to practices through the practice facilitators and the tool kit of resources. Decisions about which

PCOR findings to implement will be decided by practices. Practice facilitators will help practice leaders to understand the evidence strength, potential for impact, and feasibility as they consider what to implement. Throughout the study, we plan to share newly identified PCOR findings with other grantees. At the start of the study and quarterly, the research team will scan for new PCOR findings from multiple sources including:

- AHRQ Effective Health Care Program (http://www.effectivehealthcare.ahrq.gov/);
- Patient-Centered Outcomes Research Institute (http://www.pcori.org/);
- USPSTF (http://www.uspreventiveservicestaskforce.org/);
- Daily media monitoring reports provided internally to the USPSTF (shared with Drs. Krist and Epling);
- Quarterly LitWatch Newsletter to internally scan for new evidence about USPSTF recommendations (shared with Drs. Krist and Epling);
- National Institute on Alcohol Abuse and Alcoholism (NIAAA);
- National Institute on Drug Abuse;
- PubMed; and
- Study consultants and participants.

Practice Intervention – Process and Operational Changes

The overall intervention practices are being asked to implement is depicted in Figure 7. This is consistent with the USPSTF recommendation and is hopefully something practices are trying to do now, although we know it is poorly done. The intervention involves systematically implementing screening, counseling, and treatment for unhealthy alcohol, including SBI, stepped care, MAT, and SBIRT.

FIGURE 7. Screening, Counseling, and Treatment for Unhealthy Alcohol in Primary Care: Relationship Between SBI, Stepped Care, MAT, and Community Referral

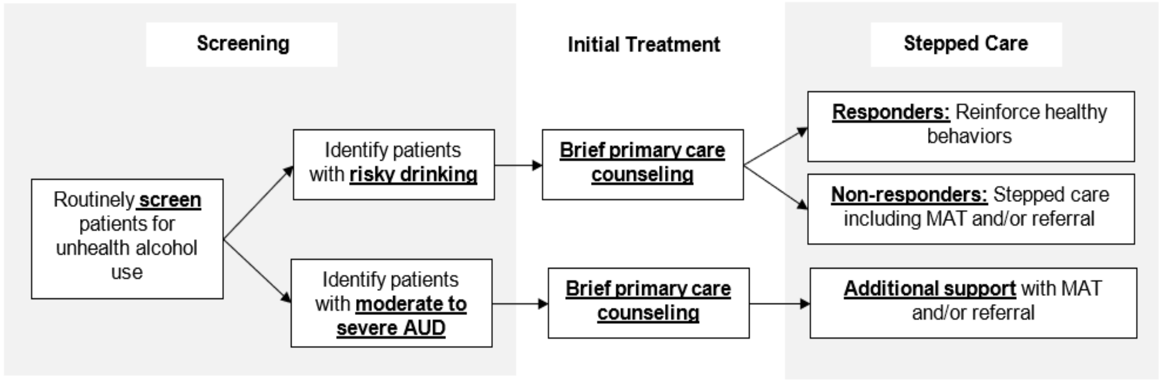

To support implementation of the intervention, each practice will be asked to make seven high leverage changes:

1. Form a quality improvement team. At the start of the intervention, each practice will be asked to assemble their Practice Team consisting of a clinician, nurse, and administrator champion. Practice Team members will meet monthly with the practice facilitator to design, carry out, and adapt their practice's intervention over time. The Practice Team will conduct the practice assessment (#2), help prepare and conduct the educational sessions (#3), develop the initial screening and counseling processes (#4, #5), develop to document care and measure performance (#7). As described below, the Practice Team will be supported throughout this process.
2. Assess practice capacity, knowledge, workflow, and needs. The first activity that the Practice Team will do with support from the practice facilitator is to assess their current screening and treatment practices, infrastructure and needs (Table 9). Practice facilitators will walk the Practice Team through this checklist and Practice Team members will survey their practice clinicians on current practice, confidence, and perceived needs. This will not only generate robust generalizable information about the current state of alcohol screening and treatment in primary care but guide the supports the Practice Team needs from the screening and counseling toolkit.
3. Attend educational sessions. Prior to starting the intervention, all clinicians will be asked to attend three thirty-minute educational sessions at the practice. The first session will be led by the regional-residency lead or Drs. Krist or Epling to review the USPSTF recommendation including the two recommended screening instruments, evidence-based counseling interventions, and roles of SBI, SBIRT, and MAT. The second session will be led by the practice facilitator to train participants in motivational interviewing

to counsel for risky drinking. Practice facilitators will all be trained in teaching motivational interviewing by Dr Svikis at the beginning of the project. The final session will be led by the practice champions to share and engage the entire practice in the planned approach for screening, counseling, and MAT. Three and six months after starting the intervention, there will also be a session to share assessment and feedback data and discuss how the practice intervention is going. These sessions will be audio recorded.

**Table 9. Initial Practice Screening and Treatment Intake Assessment**	
Screening assessment	• What is current screening practice?<br>• Is AUDIT-C or SASQ integrated into EHR?<br>• How do they document screening in EHR?<br>• Can they generate screening rate measures?<br>• Can they identify who is due to be screened?<br>• Can they send patients screening questions through the portal?<br>• Who would best do the screen?<br>• When is it best to screen?<br>• What additional supports do they need?
Treatment assessment	• What is current counseling practice?<br>• What is current MAT practice?<br>• What community and behavioral health supports are available?<br>• What patient self-management material is available?<br>• What is clinician confidence with brief counseling?<br>• What is clinician confidence with MAT?<br>• What help do they need with clinical-community and clinical-behavioral health connections?

4. <u>Commit to a process for screening.</u> Practices will be asked to commit to identifying a screening instrument and a systematic process for fielding the instrument. The practice will define a screening process, staff and clinician roles and responsibilities, screening intervals, population for screening, and method for documenting screening. Practices will be encouraged to use AUDIT-C or SASQ per USPSTF recommendations. Practice facilitators will help to guide the Practice Team in designing this process using the screening toolkit (see below for details) and then disseminating the process through practice meetings.
5. <u>Commit to a process for counseling and treatment.</u> Similarly, practices will be asked to commit to identifying a systematic counselling and treatment process for patients identified with risky drinking and AUD. The process should include an SBI, SBIRT, and MAT component; and it should include staff and clinician roles, appropriate populations interventions, follow-up, and documentation. Practice facilitators will guide the Practice Team in developing these processes using the counseling and treatment toolkit; and clinicians will receive support including motivational interviewing training, educational consults as needed, counseling support materials, educational webinars (see below).
6. <u>Identify community referral connections.</u> To support care for patients with moderate to severe AUD or who fail initial SBI as part of stepped care, practices will be asked to develop a network for community referral connections. The research team and practice facilitators will develop initial lists of community resources, in partnership with our CSBs, the NIA Clinical Trials Network, and health systems. Practice facilitators will work with the Practice Teams to refine these connections and to disseminate potential connections across regions based on prior experiences. Practice Teams with practice facilitator support will identify high yield resources, develop connection processes, and establish working relationships with community partners.
7. <u>Develop strategy to record care and measure performance.</u> As described in #4 and #5 part of developing the screening, counseling, and treatment processes will be to develop agreed upon documentation of care. This will be informed by existing infrastructure such as EHR and portal capability, planned resources for screening and counseling (e.g., use of MyPreventiveCare, My Own Health Report, or other tools), and existing workflow. Practices will also explore whether their EHR can provide screening, counseling, and treatment reports or registries of patients overdue for care. <u>*Note – we do not believe that most existing EHRs can measure performance and identify patients in need of screening and care for unhealthy alcohol use.*</u> Accordingly, we will rely on patient surveys and chart reviews for outcomes measures. However, during the intervention phase in each region, we will seek to identify and develop methods for practices to manage their own assessment and feedback using the EHR, manual review (as we will employ), or possibly the research team provided APCD reports (see below for details). All of these methods can be validated against the research collected patient surveys and chart reviews to identify sustainable methods. Then in the control phase, both the intervention and control practices will be asked to manage their own performance measurement and we will track and monitor feasibility. This will be an important generalizable finding from our study.

Practice Support Strategies (Practice Facilitation)

We propose to support practices with the above implementation activities through practice facilitation. Our practice facilitation intervention is based on the AHRQ how to guide, "Developing and Running a Primary Care Practice Facilitation Program: A How to Guide," the EvidenceNow practice facilitator toolkit, and informed by our experiences providing practice facilitation in EvidenceNow and other ACORN studies. Per the EvidenceNow revised definition of practice facilitation, our support strategies are designed to motivate, guide, and support practices in adopting, implementing, and sustaining evidence-based changes and quality improvements for unhealthy alcohol use. Our practice facilitation consists of 11 key components:

1. Provision of a practice facilitator. The facilitator will be responsible for convening and supporting each practices' change management process. Activities will include helping to form or convene practice quality improvement team; guiding each practices' intake assessment; helping to implement and manage the change process; connect practices to resources through the screening and counseling and treatment toolkits; promote reflection, strategic planning, and goal setting during Practice Team meetings and practice wide assessment and feedback sessions; and holding practices accountable to implementation and assessment activities. Given our regional rollout, each of our 4.5 practice facilitators will manage between 8 and 16 practices (depending on number of regions engaged), only 5 of which will be in the more intensive stage of designing their intervention (see Timeline below). Practices will serve as a bridge between the research team and practices, coordinating or carrying out many of out the below activities. Practice facilitators will also be responsible for collecting field notes used in outcomes assessments (see Data Sources below).
2. Engage leadership. The Practice Team will serve as local leadership. Practice facilitators will directly coach, empower, and support Practice Team activities.
3. Financial and business support. While practices will only directly receive reimbursement to collect research outcomes data, the research team will develop strategies such as guides for coding and billing to be reimbursed for screening and counseling.
4. Provision of education and training. As described above the research team and practice facilitator will conduct several practice wide educational and training sessions. Additional tools, including webinars and handouts, will be maintained in the screening and counseling and treatment toolkits. Practices will also be able to request expert consultations at any point of time to help solve any challenges; practice facilitators will also monitor practices and suggest consultations as deemed appropriate.
5. Coordination of shared learning and best practices. The first, third, and last month of each regions the intervention phase (and again for the wait list control intervention phase), the practice facilitator will coordinate a learning collaborative to share plans, experiences, and resources across practices. Learning collaboratives will be led by the practice facilitators, attended by regional-residency leads, one or more member from each Practice Team, and select members of project leadership. Meetings will be virtual using Zoom for video conferencing. Collaboratives will provide an opportunity for peer-to-peer learning and to understand local context and resources. Learning collaboratives will be recorded. Lessons learned from each region will be shared with subsequent regions.
6. Maintenance of an online support center. We will manage an online support center, originally created in EvidenceNow, where participants can find and share announcements, ideas, insights, and promising practices for practice improvement. The support center can be used by any practice member and by practice facilitators when providing practices support. Online resources will include articles, tools, tutorials, and webinars on relevant topics, plus data on community and practice measures. Information in the support center will be shared and utilized repeatedly and across regions and with other grantees.
7. Creation of a change package. To help support practices we will create a screening toolkit and a counseling and treatment toolkit. Toolkits will be maintained in the online support center. The screening toolkit will include (a) an EHR compendium of "How to screen" (assembled from current EHR in ACORN and expanded from practice intake assessments), (b) text and instructions for sending screening messages through practice portals prior to visits, (c) paper AUDIT-C and SASQ questionnaires that practices can print, (d) access to electronic screening tools (e.g. My Own Health Report, and TAPS) that can be emailed to patients prior to visit or that rooming staff or clinicians can use to guide screening and basic counseling, (e) access to an application built into practice portals, MyPreventiveCare, that can both screen and provide basic counseling for patients, and (f) guides on screening workflows and protocols. The counseling toolkit will include (a) patient educational handouts, (b) patient educational links (e.g. NIAAA Alcohol Treatment Navigator), (c) text with resource links that can be incorporated into after care summaries and patient portal messages, (d) access to electronic counseling tools (e.g.

MyPreventiveCare, My Own Health Report, Health CHEQ, etc.), (e) a compendium of potential community connections, (f) scripts to guide motivational interviewing, and (g) MAT prescribing guides. As practices identify more screening, counseling, and treatment needs and as we continually scan for more resources, we will add to the toolkits.

8. Creation of a practice facilitator roadmap. This roadmap will serve as guide and tracking system for use by practice facilitators. It will help practice facilitators ensure that practices are making the seven high leverage changes described above. The practice facilitators will create their roadmap at the start of the study. It will be based on the administrative tools in the EvidenceNow Tools for Practice Facilitators including “Documenting Your Work with Practices,” “Clinic Intervention Tracking Sheet,” and “Practice Progress Notes Template.”
9. Provide assessment and feedback. As described in detail in Section 4, participating practices are expected to assist with collecting and reporting on multiple dimensions of their experience. Included are implementation, screening, counseling, and MAT metrics. Practices will collect these metrics at baseline, month 3, and month 6 of the intervention practice fielding. VCU will analyze results and provide it back to practices referenced against the experience of others in their region, other regions, and nationally. As described above, as wait list control practices enter the intervention phase, we will test practices collecting their performance measurements through learned strategies.
10. Additional Supports. Throughout the study, practice facilitators will be encouraged to tailor the support activities to match the practice’s needs. We anticipate some practices will need more intensive support through the Practice Team, learning collaborative, direct practice facilitation, toolkit, and expert consultations. Others will mainly need direction on the USPSTF recommendation and tools for screening and counseling. If needed, practice facilitators will arrange for additional supports (e.g. informatics consultation, community connection support, MAT training). As practices complete the intensive 3-month implementation phase of their intervention, they will be transitioned to maintenance support primarily through the online learning center and email/video check ins with their practice facilitator.

Challenges and Mitigation Strategies

We learned several key lessons from EvidenceNow and we have adapted this study’s design accordingly. Specific challenges and how this study address them include:

- Challenges accessing EHR data – both because of the EvidenceNow challenges and limited querying functionality for alcohol screening, counseling, and treatment, we will not rely on EHR queries for data. Instead we will guide practices to do chart review and survey patients and develop strategies for practices to self-monitor performance during the first intervention phase in each region.
- Practice drop out – In EvidenceNow, we had about 15% of practices dropout or not submit outcomes data. We will over-recruit (n=150) to ensure 125 practices participate. To ensure participation, we have a simplified data acquisition process and practices need to deliver baseline data prior to randomization.
- Low intensity practice facilitation – practice facilitation in EvidenceNow was relatively low intensity. Facilitators often interacted with only a limited number of staff and interactions were less structured. For this study, the staged rollout across five regions will allow more intensive practice facilitation. The Practice Team will also augment the practice facilitator and educational activities will include all practice clinicians.

Additional potential challenges and barriers and how we propose to address them are as follows:

- Recruitment and training of practice facilitators – existing practice facilitators with extensive experience will hire, train and co-facilitate three new facilitators using tools developed in EvidenceNow.
- Practice recruitment – we will make use of existing relationships with partner organizations, offer plausible value proposition, use relatively simple study design strategies to make it easy for practices to participate.
- Competing demands – we will provide practices with tools to help them to deliver evidence-based care. Ideally, this will make it easier for clinicians to care for patients and increase confidence in their ability.
- Patient survey responses – because the alcohol use and treatment are sensitive questions that may impact survey response, we will add additional health behavior and clinician satisfaction questions. The survey will be pitched as a practice QI initiative and responses will be anonymous and not seen by the clinician, but rather used to improve the care delivery process.

Disadvantaged Populations

We plan to recruit practices that represent the full spectrum of primary care, including FQHCs and safety net practices. ACORN has a long track record of conducting research to help disadvantaged populations. Many of our practices care for uninsured patients and patient with Medicaid. In fact, the VCU health system cares for

nearly 50% of Virginia's uninsured patients. Likewise, many of our practices have high proportions of ethnic and racial minorities, particularly African American patients. Inclusion of the VirginiaTech Carilion regional hub will further add more rural practices that have a different set of disadvantages in terms of alcohol use and accessing care. Throughout recruitment, we will ensure that each region includes at least 5 practices that care for a more disadvantaged population. When developing our screening and counseling toolkit, we will ensure the inclusion of resources that can be used to care for more disadvantaged populations (e.g. access to free services, tools to address social needs, etc.). Finally, when we analyze our outcomes, we will conduct subgroup analyses to determine if age, race, ethnicity, insurance status, education, income, comorbidities, or rurality/urbanicity are associated with differences in screening or treatment.

Sustaining SBI and MAT

Our intervention is designed to sustain improvements in SBI and MAT within the study practices and performance measurement. Maintaining these activities is not dependent on research funding. Once trained and provided with tools for screening, counseling, and treatment, clinicians should be able maintain improvements. The connections created between the practices and residencies, CSBs, and other community support programs should be sustainable through ongoing collaborative care of patients. The motivational interviewing skills clinicians learn can be applied to other clinical situations.

Sustaining Infrastructure

This project will continue to sustain infrastructure created through EvidenceNow and it will integrate this infrastructure into the ACORN network. Infrastructure, including the added practice facilitators, will continue to be sustained through ongoing ACORN work. Additionally, many elements of this study's infrastructure (e.g. clinical-community connections, toolkits, practice-residency connections) will be sustained by our partners. DMAS and DBHDS will promote behavioral health services as part of Virginia's continued Medicaid expansion efforts. And infrastructure elements can be used to support the VCU Wright Center's community engaged research efforts, VCU's Clinical Translational Science Award funded center (Dr. Krist is the Director of Community Engagement).

Section 4: Evaluation Plan

Data Sources

We propose a robust internal mixed methods evaluation that will measure our three specific aims. We also plan to collaborate with AHRQ, the evaluator, and other grantees. Accordingly, we will collect and share with AHRQ and the evaluator all required indicators. We will use eight data sources to address the questions in our three specific aims: practice facilitator field notes, chart reviews, patient survey, clinician survey, Practice Team survey, APCD data, audio recording, and qualitative interviews (Table 10).

<table>
<tr><th colspan="3">TABLE 10. Overview of Data Collection Methods and Analysis</th></tr>
<tr><th>Aim</th><th>Data sources</th><th>Analysis</th></tr>
<tr><td>Aim 1 - Screening: To evaluate whether practice facilitation increases screening rates for unhealthy alcohol use in primary care</td><td>• <u>Chart review</u> to determine if patient's alcohol use is documented and if patient was screened; and to identify co-factors influencing outcomes
• <u>Patient survey</u> to determine if patient was asked about alcohol use; to assess if the patient is a risky drinker; and to identify co-factors influencing outcomes</td><td rowspan="2">• (Effect) Increase in screening/treatment at 3 months for intervention versus wait list control practices (generalized linear mixed model)
• (Health outcome) Change in alcohol use for patients receiving any treatment versus not (generalized linear mixed model)
• (Maintenance) Screening/treatment rate at 6 months versus 3 months (generalized linear mixed model)</td></tr>
<tr><td>Aim 2 - Treatment: To evaluate whether practice facilitation increases treatment for unhealthy alcohol use in primary care</td><td>• <u>Chart review</u> to determine if any treatment for alcohol use was documented; and to identify co-factors influencing outcomes
• <u>Patient survey</u> to determine if patient was counseled or treated for alcohol use; to identify co-factors influencing outcomes; to assess reduction in alcohol intake</td></tr>
<tr><td>Aim 3 - Implementation: To understand the practice facilitation and implementation strategies that influence effectiveness</td><td>• <u>Clinician survey</u> to assess practice patterns and confidence with alcohol screening and treatment
• <u>Practice Team survey</u> to assess practice's adaptive reserve and change capacity
• <u>Practice facilitator field notes</u> to understand what was done and by whom to implement each practice's intervention
• <u>Semi-structured interviews</u> with practice facilitators, clinicians, and stakeholders to understand intervention implementation</td><td>• Identification individual, practice, health system/community, QI processes, and intervention factors that influence intervention success (analysis and deductive approach using CFIR coding framework)
• Patient, practice, and implementation factors that influence screening/treatment (generalized linear mixed model)</td></tr>
</table>

Practice facilitator field notes. Throughout the study, practice facilitators will keep detailed field notes for every interaction. Field notes will include who participated, type of interaction, what happened during interaction, practice progress, and decisions made. Any email interactions will be saved in their entirety. For planned interactions (e.g. Practice Team meetings, academic detailing sessions, screening and counseling needs intake assessment), we will develop structured templates to ensure practice facilitators record needed data elements. Each week, practice facilitators will also record general thoughts and progress for each practice in the planning and intervention phases. All field notes will be entered into RedCap using a blend of structured (e.g. attendees, type of interaction, length of interaction) and unstructured fields (e.g. challenges addressed, decisions made, perceived engagement of attendees). Collectively, these detailed and organized notes will be used understand each practices' implementation strategy, who from the practice was engaged, and how engaged practice members were.

Clinician survey. All practice clinicians will be asked to complete a survey on paper or through RedCap (whichever each clinician prefers) to assess their profile (e.g. age, gender, race/ethnicity, degree, years in practice, FTE, etc), confidence with screening and counseling for unhealthy alcohol use, understanding of the USPSTF guideline, knowledge of practice strategy for screening and counseling, attitudes towards screening and counseling, perceived challenges and barriers to screening and counseling, and the Maslach Burnout Inventory. The survey will be administered just prior to starting the intervention and again at the end of the maintenance period (6 months after going live).

Practice Team survey. During the initial Practice Team meeting, the practice facilitator will guide the team to complete the three question adaptive reserve or Practice Culture Assessment (PCA) and the Change Process Capacity Questionnaire (CPCQ). This will be repeated at the end of the maintenance period as well. Responses will be used to help inform the practice facilitator as to specific practice needs for an effective intervention implementation. Additionally, responses will be used as part of our implementation analysis to understand factors associated with more effective implementations.

Chart review. At baseline, 3 months, and 6 months, practices will be asked to do a chart review on 60 patients. Practice facilitators will train, coordinate, and assist practices in doing this task if needed. Patients for chart review will be randomly selected from patients seen for an office visit in the prior month time period. Patients age 18-75 will be included. Acutely ill patients will be excluded (defined as any patient sent to the emergency room or admitted to the hospital from their office visit). To generate the sample, practices will be asked to generate a list of all unique patients seen in the prior month. Using a random number generator, the research team will let the practice know which patients to include based on numerical ranking. Chart review data will be used to assess outcomes for aims 1 and 2, provide contextual data for aim 3, and additional data needed for evaluator/AHRQ analyses. Using a structured RedCap template, chart abstracters will record for each study patient: a practice assigned patient study ID, age, sex, race-ethnicity, insurance type, preferred diagnosis, active diagnoses, documented alcohol use (whether documented and what is documented), documented screening (whether screened, when screened, how screened), documented counseling (when and content of counseling), provision of MAT (when and what), referral to treatment programs (when, program, and if follow-up documented), and additional documented health behaviors.

Patient survey (screening-treatment). We will also mail the chart review sample of patients a postal survey. Given that there is generally poor documentation of alcohol screening and counseling, we believe that this survey will be critically important to understand not only the baseline screening and treatment rates, but also screening and treatment during the intervention period (including both missed screening and treatment for patients with risky drinking and undocumented screening and treatment – although the hope is the intervention reduces the latter). Patient survey data will be used in conjunction with the chart review data to assess outcomes for aims 1 and 2 and provide contextual data for aim 3, subgroup analyses, and evaluator/AHRQ analyses. For each survey, we will optimize the response rate by using a modified-Dillman method. Practice facilitators will help each practice to mail surveys on practice stationery and in practice envelopes, including a personal note from the patient's clinician. The survey return envelope will be addressed to the VCU research team for data entry and analysis. Surveys will include the practice assigned patient ID to link survey responses to the chart review. Only practices will be able to link the patient ID with the patient and they will not receive raw data. VCU will receive responses, but not be able to link responses to any patients. Surveys will ask patients basic demographic information not in the chart (education, income, marital status), whether their clinician has asked them about alcohol use in the past year, the AUDIT-C questions, whether their clinician has counseled them about healthy drinking levels or advised them to reduce the amount they drink, and whether they have been given any educational materials to help reduce alcohol use. The surveys will include some additional questions about other health behaviors (e.g. diet, exercise, tobacco use) and the primary care

Measures that Matter being developed by the Larry Green Center and the American Board of Family Medicine through the Starfield III summit. These additional questions would be added to avoid only including sensitive questions on the survey (e.g. alcohol use) and to provide practices valuable information about their practice and patients that they would not otherwise collect. Final additional questions not related to study outcomes will be further refined by practices.

Patient survey (health outcome). Patients who have positive AUDIT-C screen on postal survey or have documented unhealthy alcohol use on chart review will receive a follow-up survey 6 months later to reassess alcohol use, screening, counseling, and treatment. The VCU research team will send practices the list of patient IDs to re-survey and the practice facilitator will assist and coordinate sending this follow-up health outcomes survey using the same modified-Dillman methodology for the original survey. Responses will be used to calculate whether risky drinkers have received subsequent screening, counseling, treatment, or follow-up; the proportion of risky drinkers who make improvements; whether risky drinkers used any educational materials or followed up on referrals; and the association between receiving primary care screening and counseling with any improvements.

APCD data. As part of our Medicaid expansion evaluation, we have access to statewide APCD data. The APCD includes medical and pharmacy claims submitted by commercial and public insurance carriers for over 5 million of Virginia's 8.4 million residents. All submissions include institutional encounters (practice and clinician name), medical professional services (diagnoses, counseling claims) and pharmacy services (MAT prescriptions). We will use this data to calculate diagnosis of unhealthy alcohol use and AUD, frequency of counseling, frequency of MAT by practice and clinician. This will augment our assessment and feedback data, outcomes assessment, and possibly serve as a long-time mechanism for monitoring practice performance.

Audio recordings. The practice assessment and feedback meetings and the regional learning collaboratives will be audio recorded. Recordings will be used to augment field notes to understand how practices implemented their intervention.

Semi-structured interviews and site visits. We will identify a sub-set of eight practices from each regional cohort for participation in interviews – four high performing practices and four lower performing practices (total n=40). Semi-structured interviews will be conducted with each of the three Practice Team members at the end of the maintenance period (six months after starting the intervention). Interviews will longitudinally assess domains found within CFIR and PARIHS, including practice champions' knowledge and perceptions of unhealthy alcohol use and the role of primary care in addressing it; experiences implementing SBI and MAT for unhealthy alcohol use and working with the practice facilitators; multi-level contextual factors influencing implementation, including local, organizational and health system characteristics; what concrete practices change made; how widely spread throughout their practices these changes were made; and how they accomplished this widespread change. Most interviews will be conducted over the phone, digitally recorded, and transcribed verbatim. One high performing and one low performing interview per region (n=10) will be conducted in person and include a site visit to help inform the analysis with observations. Interviews will be used with the practice facilitator notes to evaluate aim 3.

Outcomes

Table 11 provides a list of planned outcome measures and how we propose to define them. We will update these definitions at the start of the study based on input from the evaluator and AHRQ if needed. The definitions span the outcomes in our proposed aims and all required indicators specified in the RFP.

**TABLE 11. Proposed Outcome Measure Definitions**			
**Domain**	**Measure**	**Definition**	**Source**
Implementation metrics	Practice support	Number and types of personnel working with practices to support implementation	Facilitator field notes, champion interviews, site visits, audio recordings
		Number, duration, and mode of interactions between project staff/consultants and practices	
	Practice interventions	Type and quantity of strategies implemented	
		Number of practices fielding each strategy	
	Clinician engagement	Number of clinicians attending each activity	
		Percent of clinicians aware of practice strategy for screening and treatment	Clinician survey
Screening metrics	Screening rate	Percent of patients screened in each practice using:<br>- Any method<br>- Using a validated tool (AUDIT-C, SASQ)<br>- Reported by patient<br>- Documented in chart	Chart review, patient screening-treatment survey, APCD data
Treatment metrics	Treatment rate	Percent of patients with risky drinking who receive treatment using:	

		- Any treatment<br>- Brief counseling<br>- MAT<br>- Referral to community program<br>- Reported by patient<br>- Documented in chart	
Health outcome metrics	Prevalence	Percent of patients with risky drinking including<br>- AUDIT-C>4 (3 for women)<br>- Any binge drinking<br>- Drinking more than weekly recommendation	
	Reduced drinking	Percent of patients who reduce drinking at 6 months including<br>- Lower AUDIT-C score<br>- Reduced binge drinking<br>- Reduced number drinks per week	Patient health outcome survey
Note – from the data on the random sample of 60 patients, results will be extrapolated to the entire practice population			

Quantitative Analytic Plan

The generalized linear mixed model framework will be used for analysis, which will account for the nesting of patients within practices. These models will include (separately) as patient-level outcomes binary indicators of screening for alcohol use and documented evidence of screening using Audit-C or SASQ (Aim 1), treatment (Aim 2a), and MAT use (Aim 2b). Each model will include a two-level fixed group effect (intervention vs. control), and three-level fixed time effect (baseline, 3 months, 6 months), a group-by-time interaction, and a practice-level random effect to account for clustering of patients within practices. We will compare rates of change between intervention and control practices from baseline to 3 months, and baseline to 6 months.

Sample Size Determination

The following power calculations are based on Murray (1998) and Donner and Klar (2000), to account for the varying treatment effectiveness between clinicians due to (i) clinician-based randomization, and (ii) nesting of patients within clinicians. We plan to solicit surveys from 60 patients from each of 125 practices (60 intervention; 60 control). Assuming a 40% non-response rate, then ~25 surveys from each practice (3,125 total) will achieve 90% power (with 5% type-I error rate and intra-cluster correlation of 0.05) to detect (i) a 10% difference in screening rates (76% in control vs. 86% in intervention), and (i) 10% difference in documented screenings using AUDIT-C or SASQ (20% in control vs. 30% in intervention). Further assuming a 20% AUD rate, then 5 surveys and/or chart reviews per practice (625 total) will achieve 80% power (with 5% type-I error rate and intra-cluster correlation of 0.05) to detect (i) a 10% difference in counseling rates (20% in control vs. 30% in intervention), and (ii) a 10% difference in MAT rates (20% in control vs. 30% in intervention).

Qualitative Analytic Plan

To assess our four-practice implementation and support questions (how practice implementations strategies; how practice facilitation factors; how community, organization and practice-level factors; and how adapt implementation strategies influence intervention success), we will conduct a mixed methods analysis using a well described methodology guided by the CFIR framework. Our analysis will use the semi-structured interviews from the 20 higher and 20 lower performing practices and a more general analysis based on practice facilitator field notes for all 125 practices. All qualitative data will be managed using qualitative database software, Atlas.ti. A subset of the research team, led by Dr. Brooks, will read through the full dataset several times to identify main content areas. From the field notes, the team will derive the type and intensity of support provided by facilitators (e.g. practice facilitator, academic detailing, shared learning, screening and counseling toolkits, data support, and audit and feedback), and reach (i.e. the extent to which facilitation benefits the practice care team). To analyze the semi-structured interviews, the team will use both template-based and emergent coding techniques. They will create an *a priori* codebook in which codes are given meaningful definitions and applied in a standardized manner for template analysis and while coding emergent codes will be identified by discovering meaningful ideas I the data not represented in the predetermined code for emergent analysis. The team will then follow a protocol-driven approach to analysis that includes: 1) group reading of the data to refine *a priori* codes, identify emergent codes, and reach agreement on code definition; 2) independent *test coding*, during which a subset of documents, selected for variation, are coded to test the operational limits of the codebook and the ability of coders to apply codes reliably and consistently; and 3) independent coding combined with scheduled merges of coded data and weekly team coding huddles allow early detection of threats to inter coder reliability. Once coded, the research team will identify themes within the data. Once

themes are identified, the team will code data relevant to each theme, in addition to any disconfirming evidence or evidence in the data challenging theme significance.

Then using the CFIR as a coding framework as previously described, two independent reviewers will extrapolate a numerical rating for each CFIR domain for each practice. Based on the codes and details in the practice facilitator field notes, the team will write a case memo for each practice, organized by each CFIR construct with supporting quotes. Each practice case memo will then be subject to a rating process ranging from -2 to +2 to each construct for each practice. The ratings reflect the positive or negative influence of each construct on the study implementation. Finally, a matrix will be created that lists the ratings for each CFIR construct for each practice in relation to the study outcomes. Practices with lower improvement will be qualitatively compared and contrasted to practices with higher performance so as to identify patterns in ratings of the CFIR constructs. This will be used to develop an understanding of how clinician, practice, practice support, and practice intervention characteristics and how they are implemented influence the effectiveness of our intervention to help practices implement screening, counseling, and treatment for unhealthy alcohol use.

Section 5: Dissemination Plan

We are committed to communicating our project results and insights to multiple stakeholders and we are also committed to cooperating with AHRQ and its contractors in promoting project findings. The findings from this study will be highly relevant to VDH, DMAS, and DBHDS which are trying to improve behavioral health in Virginia, promote Medicaid expansion, and address the opioid epidemic. In addition to presenting and publishing our findings in scientific meetings and journals, we will launch a concerted outreach initiative to share our results with practices, health systems, practice-based research networks, community providers, behavioral health agencies, payers, government officials, and the public.

Our dissemination plan will be guided by AHRQ's Dissemination Planning Tool framework. Details of dissemination efforts will vary pending findings, but we anticipate the following: <u>Findings and products</u>. Findings of interest will include our primary study results, prevalence of unhealthy alcohol use and current practice in primary care, practice facilitation implementation findings, what it takes for primary care to better address unhealthy alcohol use, and types of treatments and community partners needed to address unhealthy alcohol use. Products will include papers, presentations, practice briefs, policy briefs, and webinars. We will provide our stakeholder partners with finding briefs after our midterm and final evaluations. Pending interest, we will prepare in person briefings. <u>End users.</u> Multiple individuals and organizations could benefit from access to the research findings and using the strategies and products developed through this project. We anticipate that end users will include primary care practices, health systems, behavioral health providers, state and federal policy makers, and public health agencies. <u>Dissemination partners.</u> We will work closely with our stakeholders (Table 5, above: VCHI, VAFP, ACP, VDH, DMAS, DBHDS, Virginia Hospital and Healthcare Association, Virginia Association of CSBs, NIDA Clinical Trials Network) to reach out to local and state end users. We will also work with the USPSTF Dissemination and Implementation committee, the evaluation team, and AHRQ to reach out to national end users. <u>Communication.</u> We will employ multiple communication channels to disseminate findings. Examples will include: website postings (ACORNpbrn.org, InnovateVirginia.org, dissemination partner websites), listserv updates (e.g. ACORN, VCHI, and Wright Center listservs), conference and webinar presentations, professional journals publications, press releases, media interviews, policy briefs, and social media. <u>Evaluation.</u> We will monitor dissemination efforts to ensure they are impactful using the following measures: product (number of dissemination products produced for dissemination), partnership (number of dissemination partners listed in Table 5 who actively engage in dissemination efforts), reach (number of organizations and individuals exposed to each dissemination product), and influence (actions taken by partner organizations in response to disseminated products).

Section 6: Project Timeline

For each participating practice we anticipate a 16-month timeline from recruitment to completion (Table 12). Prior to startup there will be at least a 3-month recruitment phase (see Table 13). Once recruited intervention and control practices will perform baseline data collection and assemble their practice team in the first month (this can occur in the recruitment phase prior to month 1). For intervention practices, the practice team will meet with the practice facilitator monthly in months 1 through 8. Initial meetings will be to do the intake assessment, plan for the practice wide educational sessions, and develop a practice approach to screening, counseling, and treatment for unhealthy alcohol use. Later meetings will check on progress, receive audit and feedback, troubleshoot, and help interpret how their intervention worked. Practice wide meetings will occur in months 1, 2, 3, 5, and 8. Months 1-3 will be for the educational sessions to learn about (1) the guideline, counseling, and MAT taught by the regional-

residency faculty, (2) motivational interviewing taught by the practice facilitator, and (3) the practice plan for screening, counseling, and treatment processes taught by the practice champions. The meetings in month 5 and 8 will be to receive audit and feedback and troubleshoot the practice's implementation strategy. Learning collaboratives will occur in the first month of going live, and immediately after each data collection period when practices receive their audit and feedback. Control practices will conduct similar activities but delayed to months 9-16. At any point in time, intervention and control practices can ask for an educational or support consult from the practice facilitator or regional-residency or project team faculty. Practice facilitators may also suggest consults for practices that appear to be having difficulties implementing their intervention. Both intervention and control practices will survey patients and review charts with practice facilitator support in months 5 and 8; they will then collect audit and feedback data on their own in months 13 and 16 based on lessons learned in the prior phase.

**TABLE 12. General Practice Timeline**																
**MONTH**	**1**	**2**	**3**	**4**	**5**	**6**	**7**	**8**	**9**	**10**	**11**	**12**	**13**	**14**	**15**	**16**
Baseline data collection	I/C															
Assemble Practice Team	I/C															
Practice educational sessions	I	I	I		I			I	C	C	C		C			C
Practice leads meet with facilitator	I	I	I	I	I	I	I	I	C	C	I/C	C	C	I/C	C	C
Field Intervention			I	I	I	I	I	I			C	C	C	C	C	C
Learning collaborative participation			I		I			I			C		C			C
3- and 6-month data collection					I/C			I/C								
Self-monitoring data collection													I/C			I/C
Implementation interviews									I	I						
Note: I = Intervention practices, C = Wait list control practices																

Overall Project Timeline

Compiling the five regional timelines and additional project activities, the overall project timeline is shown in Table 13. To ensure effective practice recruitment, recruitment activities for regions 2-5 will start will prior regions are conducting their study; assembling the practice team and planning for baseline data collection can occur once the practice is recruited. Baseline data collection can occur in month one or the month prior. While we will initially assemble practice and facilitator materials (e.g. data use agreement, change package, intake assessment, facilitator roadmap, screening toolkit, and counseling toolkit) at the beginning of the study, as an adaptive intervention, all materials will be updated throughout the study period based on lessons learned from prior study cohorts and regions. Potential changes will be brought to the project team by the practice facilitators during the bi-weekly team meetings. The staged regional rollout means that even during the busiest time periods, the maximum number of regions with intervention practices implementing their intervention will be 3 (n=39). However, in any quarter there will only be 1 intervention cohort going live (n=13). This means practice facilitators will be in charge of a maximum of 3 practices preparing to go live and 8 practices in the intervention phase. In the sixth quarter (busiest quarter) they may also be managing 5 control practices implementing their intervention.

**TABLE 13. Overall Project Timeline**												
**Study QUARTER**	**1**	**2**	**3**	**4**	**5**	**6**	**7**	**8**	**9**	**10**	**11**	**12**
**Development Phase**												
IRB application	X											
Prepare practice materials	X											
Hire/train practice facilitators	X	X										
Assemble/update toolkits	X	X	X	X	X	X	X	X	X	X	X	X
**Intervention Phase (Dissemination and Implementation)**												
Region 1 activities	R	I	I	I	C	C	C					
Region 2 activities	R	R	I	I	I	C	C	C				
Region 3 activities	R	R	R	I	I	I	C	C	C			
Region 4 activities	R	R	R	R	I	I	I	C	C	C		
Region 5 activities	R	R	R	R	R	I	I	I	C	C	C	
**Data Collection and Analysis Phase**												
Regional data collection		X	X	X	X	X	X	X	X	X	X	
Midpoint and final analyses						X	X				X	X
Participation in evaluator and AHRQ collaborative activities	X	X	X	X	X	X	X	X	X	X	X	X
Note: R=practice recruitment, X=scheduled activity												

To facilitate analysis, data collection will be ongoing through the practice facilitation phase and we will plan for a mid-point analysis. The mid-point analysis will allow us to refine our planned final analysis, assess mid-point progress, identify any data deficiencies, understand how the implementation is unfolding, refine the intervention, and present/publish midpoint implementation findings. Throughout the study, we will participate in all evaluator and AHRQ activities. We look forward to a shared learning process with other grantees.

DATA AND SAFETY MONITORING PLAN

Data Management Methods

To protect patient privacy and confidentiality, we will utilize protections to managing the chart review, patient survey and transcript data similar to what we have done on many prior studies. The process results in researchers who have access to personal health information not having access to any personally identifiable information, and practice staff who have access to personally identifiable information not having access to any patient reported personal health information beyond what the patient has shared with the practice. The process for this project is described in detail below.

The only patient identifier will be the medical record number (MRN), which only practices will have access to. To reduce the risk of breaches of confidentiality, we replace the MRN in the research databases with a patient ID. This key code will be used for linking chart review data with patient survey data. The practices will store the MRN to patient ID linking table separately from all other data. By compartmentalizing data access to potential identifiable data, similar protocols have been approved as posing minimal risks to patients' privacy and confidentiality.

For chart reviews, all abstracted information will be documented in HIPPA-compliant, encrypted, password protected RedCap with the patient ID as the only identifier. The file linking the MRN and patient ID will be destroyed at the end of data collection. For the protection of transcript data, any individually identifying information will be removed from the transcripts, except for a key code to link the transcripts to practices. Transcriptions and qualitative data analyses will be stored in password protected files, and recordings will be erased and/or destroyed after they are analyzed.

All data will be securely stored in a password-restricted DB2 mainframe database. Paper surveys and interview audio recordings will be physically secured in locked file cabinets until destroyed.

It is unlikely that the study will result in any adverse effect that may require medical or professional intervention. Patients who report unhealthy drinking will be encouraged to talk with the clinician about reducing their alcohol use.

Collection and Reporting of Adverse Events (AE) and Serious Adverse Events (SAE)

The main adverse event that could occur is breach of confidentiality or privacy from release of identifiable information. Any release or potential release of identifiable data will be immediately reported to the principal investigator. After determining the extent and content of the released information, the principal investigator will create an adverse event report that will be shared with Virginia Commonwealth University's IRB and the AHRQ program officer. The report will state the extent of the data breach, list the types of identifiable information that were released, describe the actual or potential causes of the release of identifiable information and list a remediation plan including all steps taken or planned to stop further release of information. The principal investigator will work with the IRB and the AHRQ program officer to determine the best course of action, including approving the remediation plan and whether to continue or discontinue the study.

Communication Plan with IRB/NIDA

The principal investigator will communicate directly with the IRB and AHRQ through designated program officers. This communication will occur via phone or email. Any adverse events will be communicated within 24 hours.

Data and Safety Monitoring Board

Because this is not a clinical trial, rather a practice facilitation intervention to promote recommended evidence-based care, we will not assemble a data and safety monitoring board.

Monitoring Data and Safety

Throughout the study process the Principal Investigator (Krist) and the lead biostatistician (Sabo) monitored all data collection efforts and adherence to study protocols including the above safety plans. This included weekly meetings with the Practice Facilitators and study team to review activities, quarterly checks on database structure and security, and annual review and update with the VCU IRB.
