# Supplementary material for: Practice Facilitation to Address Unhealthy Alcohol Use in Primary Care: A Cluster Randomized Clinical Trial
Source: JAMA Health Forum. 2024 Aug 9;5(8):e242371. doi: 10.1001/jamahealthforum.2024.2371 (PMC11316228; doi:10.1001/jamahealthforum.2024.2371)
Supplement: Supplement 2. — eAppendix. Practice Facilitator Guide eAppendix 2. Sensitivity Analysis – Screening, Counseling, and Treatment Outcomes for All Patients Aged 18 to 79 Years with a Visit Eligible for Screening [file jamahealthforum-e242371-s002.pdf]

Supplemental Online Content

Huffstetler AN, Villalobos G, Webel B, et al. Practice facilitation to address unhealthy alcohol use in primary care: a cluster randomized clinical trial. JAMA Health Forum. 2024;5(8):e242371. doi:10.1001/jamahealthforum.2024.2371

eAppendix 1. Practice Facilitator Guide

eAppendix 2. Sensitivity Analysis – Screening, Counseling, and Treatment Outcomes for All Patients Aged 18 to 79 Years with a Visit Eligible for Screening

This supplemental material has been provided by the authors to give readers additional information about their work.

© 2024 Huffstetler AN et al. *JAMA Health Forum*.

eAppendix 1.
Practice Facilitation to Promote Evidence-Based Screening and Management of Unhealthy Alcohol Use in Primary Care

Practice Facilitator Guide

Virginia Commonwealth University

Last Updated March 21, 2024

© 2024 Huffstetler AN et al. *JAMA Health Forum*.

Table of Contents

1. Study Purpose
2. Intervention Overview
3. Practice Intervention
4. Practice Facilitators
5. Practice Support Strategies
6. Practice Teams
   a. Practice Intake Form
   b. Practice Champions
   c. Practice Team Survey
7. Practice Facilitator Agendas
   a. Learning Collaboratives
   b. Practice Meetings
8. Field Notes
9. Additional Supports
   a. Unhealthy Alcohol Use Website
   b. Pamphlets
   c. External resources
10. Practice Intake Assessment
11. Practice Team Survey

© 2024 Huffstetler AN et al. *JAMA Health Forum*.

STUDY PURPOSE

Screening and counseling for unhealthy alcohol use (UAU) is one of the most poorly delivered health behavior counseling services in primary care. We believe that this is because most primary care clinicians do not understand that they can improve health outcomes by identifying risky drinking and providing brief counseling. Instead, most clinicians focus on identifying and treating moderate to severe Alcohol Use Disorders (AUD), which are more difficult to manage, require significantly more patient support, and benefit from medication-assisted therapy, practice infrastructure (electronic health record alerts and reminders, screening instruments). Additionally, resources (patient educational and self-management resources, referral network) have not been developed to support screening and counseling interventions for risky drinking.

We propose to provide practice facilitation to guide practices in developing a shared understanding of the evidence and standardized protocols for screening, counseling, and treatment of risky drinking and AUD. By the second year of the study, all practices (intervention and control) will receive the intervention (practice support to implement screening, counseling, and treatment of unhealthy alcohol). We will compare changes from baseline to 3- and 6-months post-intervention for intervention versus control practices. The baseline to 3-month comparison will assess effectiveness, while the baseline to 6-month comparison will assess maintenance.

Aim 1 (Screening): ***To evaluate whether practice facilitation increases screening rates for unhealthy alcohol use in primary care.*** From patient survey data, chart reviews, and All-Payer Claims Data (APCD), we will determine whether there is a greater increase in screening for unhealthy alcohol use at 3 and 6 months for patients in intervention practices versus waitlist control practices.

> *Hypothesis #1: Compared to control practices, 10% more patients in intervention practices will report being asked about alcohol use (increase from 78% to 86%) and 50% more patients in intervention practices than control practices will have a documented screen-using AUDIT-C or SASQ (increase from about 20% to 30%).*

Aim 2 (Treatment): ***To evaluate whether practice facilitation increases treatment for unhealthy alcohol use in primary care.*** From patient survey data, chart reviews, and APCD, we will determine:

- Sub-aim 2a: Whether there is a greater increase in counseling patients with risky drinking (more than 14 drinks per week for men, 7 drinks per week for women, or more than 3 drinks per occasion) at 3 and 6 months for intervention versus waitlist control practices.
- Sub-aim 2b: Whether there is a greater increase in medications for patients with moderate to severe AUD at 3 and 6 months for intervention versus waitlist control practices.
- Sub-aim 2c: Whether patients who report risky drinking reduce the amount they drink in 6 months.

> *Hypothesis #2: Compared to control practices, 50% more patients in intervention practices with risky drinking will report or have documented treatment (brief counseling and/or MAT) (increase from 20% to 30%).*

© 2024 Huffstetler AN et al. *JAMA Health Forum*.

***Aim 3 (Practice Implementation and Support Moderators):* To understand the practice implementation strategies and practice support factors that influence the effectiveness of the intervention in promoting routine screening for unhealthy alcohol use.** From practice facilitator field notes, facilitator interviews, and the clinician survey, we will code and qualitatively rate consolidated framework for implementation research (CFIR) constructs that influence intervention implementation effectiveness.

Sub-aim 3a What practice implementation strategies most benefit a practice's ability to implement screening, counseling, and treatment protocols to address unhealthy alcohol use?

Sub-aim 3b What practice facilitation factors influence implementation success?

Sub-aim 3c How community, organization, and practice-level factors impact implementation efforts.

Sub-aim 3d How practices adapt implementation strategies to reflect local needs.

© 2024 Huffstetler AN et al. *JAMA Health Forum*.

INTERVENTION OVERVIEW

To disseminate and implement screening, counseling, and treatment of unhealthy alcohol, we are proposing to provide practice support for practices in making operational and process changes. The combination of support and practice changes are designed to address the six key drivers identified through EvidenceNow that primary care practices need to build their capacity to implement the best evidence. How these drivers are addressed is shown in the table below. Based on evidence about why primary care practices do not provide more screening, counseling, and treatment for unhealthy alcohol use, our intervention has an emphasis on ensuring clinicians understand the preventive service and infrastructure support.

**Overview of EvidenceNow Key Change Drivers and Strategies to Address Drivers**		
**Key Driver**	**Change Strategy**	**How Provided**
Seek and implement evidence	Develop a process to search for new evidence	- Educational sessions to share evidence.<br>- Research team track and share new evidence.
Implement quality improvement	Develop an interprofessional quality improvement team	- Form a practice quality improvement team.<br>- Practice facilitator provides support.
Optimize health information systems	Develop standard documentation; determine if EHR supports measuring care	- Toolkit for EHR support.<br>- APCD to supplement monitoring implementation.<br>- Shared learning on EHR use for documenting and monitoring care.
Create care teams	Establish care teams and delineate roles	- Practice team to define screening, counseling, and treatment processes and care team roles.
Engage with patients and families	Identify patients effected by the evidence	- Define process to screen patients.<br>- Employ motivational interviewing for counseling.
Nurture leadership	Forge a vision for adapting new evidence	- Learning collaboratives to learn from other practices.<br>- Research team to disseminate identified best practices.

© 2024 Huffstetler AN et al. *JAMA Health Forum*.

PRACTICE INTERVENTION – PROCESS AND OPERATIONAL CHANGES

The overall intervention practices are being asked to implement is depicted in the diagram below. This is consistent with the USPSTF recommendation and is hopefully something practices are trying to do now, although we know it is poorly done. The intervention involves systematically implementing screening, counseling, and treatment for unhealthy alcohol, including SBI, stepped care, MAT, and SBIRT.

Screening, Counseling, and Treatment for Unhealthy Alcohol in Primary Care: Relationship Between SBI, Stepped Care, MAT, and Community Referral

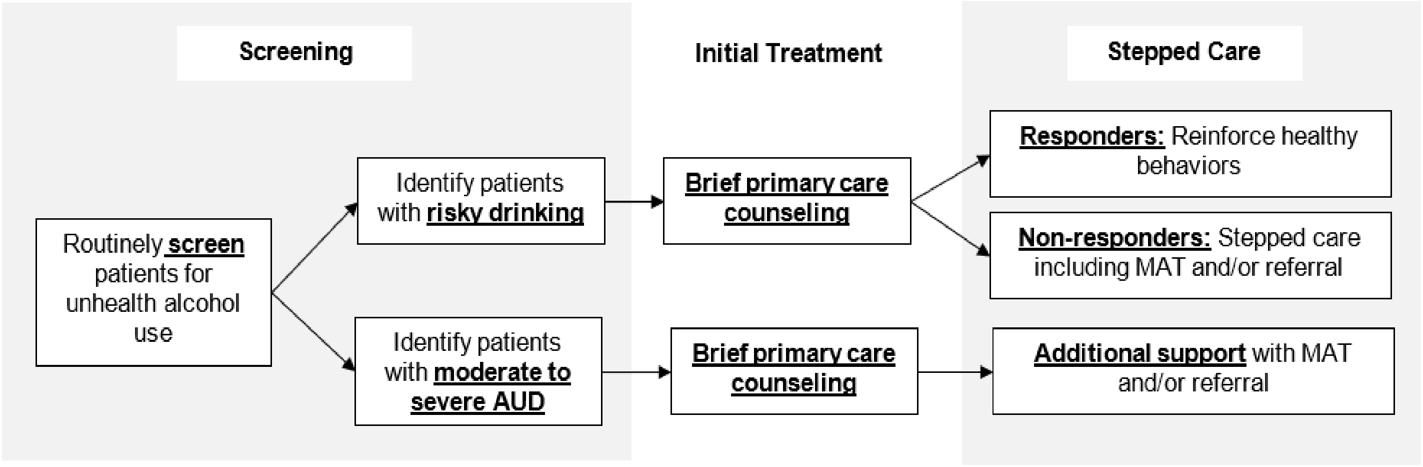

To support implementation of the intervention, each practice will be asked to make seven high leverage changes:

1. Form a quality improvement team. At the start of the intervention, each practice will be asked to assemble their Practice Team consisting of a clinician, nurse, and administrator champion. Practice Team members will meet monthly with the practice facilitator to design, carry out, and adapt their practice's intervention over time. The Practice Team will conduct the practice assessment (#2), help prepare and conduct the educational sessions (#3), develop the initial screening and counseling processes (#4, #5), develop to document care and measure performance (#7). As described below, the Practice Team will be supported throughout this process.

2. Assess practice capacity, knowledge, workflow, and needs. The first activity that the Practice Team will do with support from the practice facilitator is to assess their current screening and treatment practices, infrastructure and needs (Table 9). Practice facilitators will walk the Practice Team through this checklist and Practice Team members will survey their practice clinicians on current practice, confidence, and perceived needs. This will not only generate robust generalizable information about the current state of alcohol screening and treatment in primary care but guide the supports the Practice Team needs from the screening and counseling toolkit.

3. Attending educational sessions. Prior to starting the intervention, all clinicians will be asked to attend three thirty-minute educational sessions at the practice. The first session will be led by the regional-residency lead or Drs. Krist or Epling to review the USPSTF recommendation including the two recommended screening instruments, evidence-based

© 2024 Huffstetler AN et al. *JAMA Health Forum*.

counseling interventions, and roles of SBI, SBIRT, and MAT. The second session will be led by the practice facilitator to train participants in motivational interviewing to counsel for risky drinking. Practice facilitators will all be trained in teaching motivational interviewing by Dr. Svikis at the beginning of the project. The final session will be led by the quality improvement team to share and engage the entire practice in the planned approach for screening, counseling, and MAT. Three and six months after starting the intervention, there will also be a session to share assessment and feedback data and discuss how the practice intervention is going. These sessions will be audio recorded.

Table 9. Initial Practice Screening and Treatment Intake Assessment

Screening assessment	• What is current screening practice?<br>• Is AUDIT-C or SASQ integrated into EHR?<br>• How do they document screening in EHR?<br>• Can they generate screening rate measures?<br>• Can they identify who is due to be screened?<br>• Can they send patients screening questions through the portal?<br>• Who would best do the screen?<br>• When is it best to screen?<br>• What additional supports do they need?
Treatment assessment	• What is current counseling practice?<br>• What is current MAT practice?<br>• What community and behavioral health supports are available?<br>• What patient self-management material is available?<br>• What is clinician confidence with brief counseling?<br>• What is clinician confidence with MAT?<br>• What help do they need with clinical-community and clinical-behavioral health connections?

4. <u>Commit to a process for screening.</u> Practices will be asked to commit to identifying a screening instrument and a systematic process for fielding the instrument. The practice will define a screening process, staff and clinician roles and responsibilities, screening intervals, population for screening, and method for documenting screening. Practices will be encouraged to use AUDIT-C or SASQ per USPSTF recommendations. Practice facilitators will help to guide the Practice Team in designing this process using the screening toolkit (see below for details) and then disseminating the process through practice meetings.

5. <u>Commit to a process for counseling and treatment.</u> Similarly, practices will be asked to commit to identifying a systematic counselling and treatment process for patients identified with risky drinking and AUD. The process should include an SBI, SBIRT, and MAT component; and it should include staff and clinician roles, appropriate populations interventions, follow-up, and documentation. Practice facilitators will guide the Practice Team in developing these processes using the counseling and treatment toolkit; and clinicians will receive support including motivational interviewing training, educational consults as needed, counseling support materials, educational webinars (see below).

6. <u>Identify community referral connections.</u> To support care for patients with moderate to severe AUD or who fail initial SBI as part of stepped care, practices will be asked to

© 2024 Huffstetler AN et al. *JAMA Health Forum*.

develop a network for community referral connections. The research team and practice facilitators will develop initial lists of community resources, in partnership with our CSBs, the NIA Clinical Trials Network, and health systems. Practice facilitators will work with the Practice Teams to refine these connections and to disseminate potential connections across regions based on prior experiences. Practice Teams with practice facilitator support will identify high yield resources, develop connection processes, and establish working relationships with community partners.

7. Develop strategy to record care and measure performance. As described in #4 and #5 part of developing the screening, counseling, and treatment processes will be to develop agreed upon documentation of care. This will be informed by existing infrastructure such as EHR and portal capability, planned resources for screening and counseling (e.g., use of MyPreventiveCare, My Own Health Report, or other tools), and existing workflow. Practices will also explore whether their EHR can provide screening, counseling, and treatment reports or registries of patients overdue for care. *Note – we do not believe that most existing EHRs can measure performance and identify patients in need of screening and care for unhealthy alcohol use.* Accordingly, we will rely on patient surveys and chart reviews for outcomes measures. However, during the intervention phase in each region, we will seek to identify and develop methods for practices to manage their own assessment and feedback using the EHR, manual review (as we will employ), or possibly the research team provided APCD reports (see below for details). All of these methods can be validated against the research collected patient surveys and chart reviews to identify sustainable methods. Then in the control phase, both the intervention and control practices will be asked to manage their own performance measurement and we will track and monitor feasibility.

© 2024 Huffstetler AN et al. *JAMA Health Forum*.

PRACTICE FACILITATORS

Practice Facilitators are trained individuals who work with primary care practices, using evidence-based findings to build up the practice's capacity to ultimately improve patient care.

Practice facilitators work closely with all practice staff and clinicians, first learning about each specific practice to understand why certain processes are in place. Perspectives from different members are important.

What are some of the things practice facilitators provide?

- Education
- Guidance
- Support
- Motivation
- Access to resources
- Accountability

© 2024 Huffstetler AN et al. *JAMA Health Forum*.

PRACTICE SUPPORT STRATEGIES

Our practice facilitation intervention is based on AHRQ's, "Developing and Running a Primary Care Practice Facilitation Program: A How to Guide," the EvidenceNow practice facilitator toolkit, and informed by our experiences providing practice facilitation in EvidenceNow and other ACORN studies. (see below for links to these resources).

Our practice facilitation consists of 10 key components:

1. Provision of a practice facilitator. The facilitator will be responsible for convening and supporting each practice's change management process. Activities will include helping to form or convene practice quality improvement team; guiding each practice's intake assessment; helping to implement and manage the change process; connecting practices to resources through the screening and counseling and treatment toolkits; promoting reflection, strategic planning, and goal setting during Practice Team meetings and practice-wide assessment and feedback sessions; and holding practices accountable to implementation and assessment activities.

2. Engage leadership. The Practice Team will serve as champions for their practice. Practice facilitators will directly coach, empower, and support Practice Team activities.

3. Financial and business support. The practice facilitators will develop strategies such as guides for coding and billing to be reimbursed for screening and counseling.

4. Provision of education and training. The practice facilitators will conduct several practice-wide educational and training sessions. Resources will include webinars, handouts, and expert consultations.

5. Coordination of shared learning and best practices. The practice facilitators will coordinate a learning collaborative to share plans, experiences, and resources across practices.

6. Maintenance of an online support center. We will manage a website (uauvirginia.squarespace.com) accessible to participants for information on practice improvement. The website will be continuously updated. Online resources will include articles, tools, tutorials, and webinars on relevant topics, plus data on community and practice measures. Information in the support center will be shared and utilized repeatedly across regions and with other grantees.

7. Creation of a change package. To help support practices we will create a screening toolkit and a counseling and treatment toolkit. Toolkits will be maintained in the online support center. The screening toolkit will include (a) an EHR compendium of "How to screen" (assembled from current EHR in ACORN and expanded from practice intake assessments), (b) text and instructions for sending screening messages through practice portals before visits, (c) paper AUDIT-C and SASQ questionnaires that practices can print, (d) access to electronic screening tools, (e) access to an application built into practice portals, that can both screen and provide basic counseling for patients, and (f) guides on screening workflows and protocols. The counseling toolkit will include (a) patient educational handouts, (b) patient educational links (e.g. NIAAA Alcohol Treatment Navigator), (c) text with resource links that can be incorporated into after-care summaries and patient portal messages, (d) access to electronic counseling tools, (e) a compendium of potential community connections, (f) scripts to guide

© 2024 Huffstetler AN et al. *JAMA Health Forum*.

motivational interviewing, and (g) MAT prescribing guides. As practices identify more screening, counseling, and treatment needs and as we continually scan for more resources, we will add to the toolkits.

8. Creation of a practice facilitator roadmap. This roadmap will serve as a guide and tracking system for use by practice facilitators. It will help practice facilitators ensure that practices are making the seven high-leverage changes described above. The practice facilitators will create their roadmap at the start of the study. It will be based on the administrative tools in the EvidenceNow Tools for Practice Facilitators including "Documenting Your Work with Practices," "Clinic Intervention Tracking Sheet," and "Practice Progress Notes Template."

9. Provide assessment and feedback. Participating practices are expected to assist with collecting and reporting on multiple dimensions of their experience. Practices will collect these metrics at baseline, month 3, and month 6 of the intervention practice fielding. Practice facilitators will share results with practices.

10. Additional Supports. Throughout the study, practice facilitators will be encouraged to tailor the support activities to match the practice's needs. We anticipate some practices will need more intensive support through the Practice Team, learning collaborative, direct practice facilitation, toolkit, and expert consultations. Others will mainly need direction on the USPSTF recommendation and tools for screening and counseling. If needed, practice facilitators will arrange for additional support (e.g. informatics consultation, community connection support, MAT training). As practices complete the intensive 3-month implementation phase of their intervention, they will be transitioned to maintenance support primarily through the online learning center and email/virtual check-ins with their practice facilitator.

© 2024 Huffstetler AN et al. *JAMA Health Forum*.

PRACTICE TEAMS

The makeup of practice teams will vary, in size, number of clinicians, their patients' characteristics, capacity, EHR, etc. Practice facilitators will learn about each practice by asking a member of the practice team to complete the Practice Intake Assessment and also by learning directly from practice team members during the initial meetings.

Practice Intake Assessment

The Practice Intake Form will be emailed to administrative staff early in the enrollment process for them to complete. This information will help practice facilitators learn more about their practice and the patients they provide care to, to help practice facilitators better tailor the intervention. This form can be found at the end of the guide.

Identifying Practice Champions and Quality Improvement Team

We recommend having 3 practice champions leading the process for their practice; ideally one clinician, one nurse and one administrator. However, more staff can be included for larger offices and fewer for smaller offices as they prefer.

The ask for the quality improvement team will be to:

- attend up to four, 30-60-minute learning collaborative meetings with the practice facilitator
    - Meetings can be scheduled around their availability and preference.
- share their current screening and treatment processes (Practice Team Survey)
- communicate information with their practice members
- share input from other practice members at meetings
- propose a plan for modifying their screening and treatment process to present to the larger team

Practice Team Survey

At the first learning collaborative meeting, ask the champions to complete the Practice Team Survey to understand what their current screening and treatment process is.

PRACTICE FACILITATOR AGENDAS

As mentioned, the size of the practice teams will vary. We know it may be challenging to schedule a time that works for all, without being too disruptive with clinic obligations. Practice facilitators will be flexible in scheduling meetings around each practice's needs.

We recommend the first meeting be with the practice champions to learn about their team and their current processes (see learning collaborative agenda #1). This will help tailor future meetings.

Agendas are drafted with recommended content; however, they can be modified.

Learning Collaborative Agendas

Learning Collaborative #1 Agenda

1. Introductions
    a. Prior quality improvement projects
2. Unhealthy Alcohol Use Study Overview
    a. Practice facilitator role
    b. Data collected – who will complete the chart review
    c. Practice meeting and learning collaborative – scheduling dates
3. Current Screening and Treatment Practices
    a. Complete Practice Team Survey
4. How Can I Best Help Your Practice?
5. Next Steps
    a. Next Learning Collaborative
    b. Educational Session

Learning Collaborative #2 Agenda

1. Practice Updates
2. Unhealthy Alcohol Use Website Overview
3. Updating Screening Process
    a. Review of current state
    b. Revised screening process
    c. Tools: AUDIT-C or SASQ
    d. Roles and responsibilities
4. How Can I Best Help Your Practice?
5. Next Steps
    a. Next Learning Collaborative
    b. Educational Session

Learning Collaborative # 3 Agenda

1. Practice Updates
2. Counseling & Treatment Process
3. Local Resources
4. Documentation of Care
    a. Develop workflow for screening, discuss billing for screening/counseling, and how to schedule follow-up for UAU
5. Next Steps

© 2024 Huffstetler AN et al. *JAMA Health Forum*.

   a. Next Learning Collaborative
   b. Educational Session

Learning Collaborative # 4 Agenda

1. Practice Updates
2. Revised Workflow
3. Planning for Practice Meeting
4. Future Steps
   a. Want an extra LC to troubleshoot? Reach out at ANY time
   b. Interviews x 10 practices after the study (randomly selected)
   c. If you note any issues with the website, please just send a message and we will address it ASAP.
   d. Chart Review and Patient Surveys at 3 mo and 6 mo: Planning for EMR access or someone to review.
5. Next Steps
   a. Next Learning Collaborative
   b. Educational Session

Practice Meeting Agendas

Practice Meeting #1 Agenda (Screening and Brief Interventions)

1. Introductions
2. Unhealthy Alcohol Use Study Overview
   a. Champions share progress
3. Review of USPSTF recommendations
   a. video
4. Review of Brief Interventions
   a. video

Practice Meeting #2 Agenda (Motivational Interviewing & Medications for Alcohol Use Disorder)

1. Introductions
2. Update on Practice Progress
   a. Practice-specific information on screening rates, alcohol use, and medications
3. Motivational interviewing techniques
   a. Video
4. Medication Assisted Therapy Options for AUD
   a. Video

Practice Meeting #3 Agenda (Practice's Approach)

1. Updates
2. Planned Practice Approaches
   a. Screening
   b. Counseling
   c. Referrals
   d. Medications
   e. Website Use and Resources

© 2024 Huffstetler AN et al. *JAMA Health Forum*.

FIELD NOTES

After each meeting or other type of contact with practice team members (including emails, texts, and phone calls), document the interaction with the following information:

**Practice name**	
**Enrollment status**	
**Date of interaction**	
**Facilitator**	
**Purpose of interaction**	☐ Learning collaborative<br>☐ Practice meeting<br>☐ Combined LC2/PM1<br>☐ Combined LC3/PM2<br>☐ Combined LC4/PM3<br>☐ Practice visit<br>☐ Weekly communication log<br>☐ Other<br>☐ Post intervention check in
**Interaction type**	☐ Virtual<br>☐ In person<br>☐ Phone Call<br>☐ Email communication<br>☐ Text (personal phone)<br>☐ Text (Mosio)<br>☐ No interaction<br>☐ Other<br>☐ Hybrid
**Start time**	
**End time**	
**Duration in minutes**	
**Practice members**	
How many invitees attended? (Write as # out of #.)	
**Activities:** Are any concurrent activities or projects happening in the practice impacting implementation efforts? Explain including what activity, who is participating, and the duration of the activity.	
**Adaptations:** How is the practice adapting or modifying the intervention to fit their needs?	
**Questions:** What questions has the practice asked about the intervention?	
**Challenges:** What new challenges or barriers to implementation, if any, has the practice experienced?	
**Plans:** What is the practice's plan for making progress on the intervention?	

© 2024 Huffstetler AN et al. *JAMA Health Forum*.

**Follow-up:** Was there evidence of progress made on previously established plans or challenges? Explain.	
**Suggestions:** Did the practice offer any suggestions on how to improve the intervention?	
**Engagement:** What staff are engaged in the project? Is there evidence of practice-wide engagement or is it isolated to select team members?	
If documenting a learning collaborative or practice meeting, was the meeting recorded?	☐ Yes<br>☐ No
If documenting a learning collaborative or practice meeting, does the practice facilitator recommend analysis of this transcript?	☐ Yes<br>☐ No
Additional comments	
How would you describe the practice's ability to query requested data going?	☐ Not started<br>☐ No challenges expressed<br>☐ Running into difficulty<br>☐ Unable to query<br>☐ IT pulling data<br>☐ Practice staff pulling data<br>☐ VCU staff pulling data<br>☐ Data received
Additional comments on data pulling	
Please rate your assessment of the practice's recent progress.	☐ High<br>☐ Moderate<br>☐ Low
Entered on	
Entered by	

© 2024 Huffstetler AN et al. *JAMA Health Forum*.

ADDITIONAL SUPPORTS

There are a variety of supports available for practices. As new resources are developed, more will be added. If practices request information not already available, we will do our best to put something together and/or find an existing source.

A study website is available for practice teams at https://uauvirginia.squarespace.com. This site will be updated as new information is available.

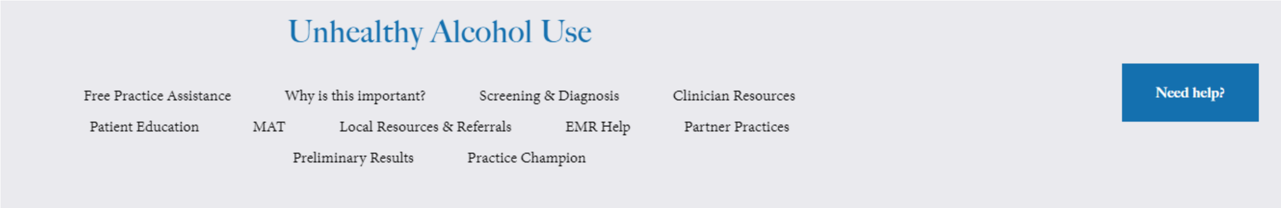

The information available on the Unhealthy Alcohol Use website includes:

- How practice teams can enroll, and what are the benefits
- Why is screening for unhealthy alcohol use important?
- Recommended screening tools and how to score them
- Interpreting results, including DSM-5 criteria
- Brief interventions including motivational interviewing
- Printable resources to offer patients
- Overview of medications available for patients with alcohol use disorder
- Local resources across Virginia for additional support for patients
- 5 brief videos (under 9 minutes each) prepared by our research team

The local resources to which patients can be referred have been verified by study team members who called each organization, speaking with staff to verify services, eligibility, cost, insurance, documentation required, and contact information. This information is printable and ready to hand to patients.

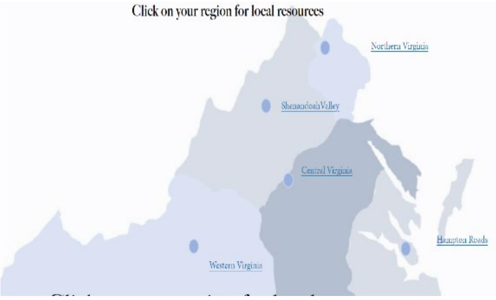

Pamphlets

Each practice will be receiving the following:

- A Clinician Guide for Addressing Unhealthy Alcohol Use (English)
- A patient pamphlet titled "Rethinking Drinking: Alcohol and Your Health (English and Spanish)

© 2024 Huffstetler AN et al. *JAMA Health Forum*.

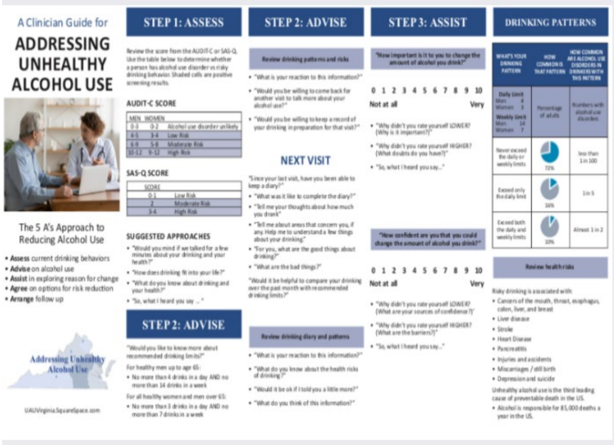

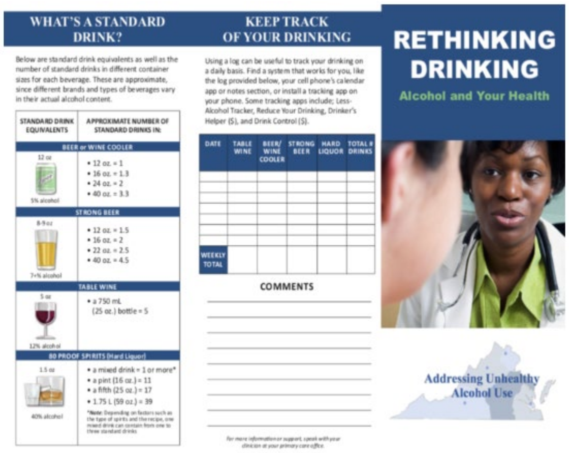

<u>Agency for Healthcare Research and Quality (AHRQ) Resources</u>

Monthly e-Newsletters on Managing Unhealthy Alcohol Use in Primary care are available on the AHRQ UAU SharePoint site

AHRQ Online Practice Facilitation Modules Available
To assist practice facilitators in gaining critical skills, AHRQ recently released a set of 14 on-demand Practice Facilitation Training Modules. These free, interactive training modules, each 20-30 minutes long, provide opportunities to practice skills and include videos of experienced practice facilitators who share insights and helpful hints. The self-directed modules, based on AHRQ's widely used Primary Care Practice Facilitation Curriculum, provide a solid foundation for new facilitators and give experienced facilitators a refresher on specific skills.

A new AHRQ handbook provides practical information and strategies for primary care practice coaches to support improved care by helping practices effectively use health information technology (IT). *Obtaining and Using Data in Practice Improvement: A Handbook for Health IT Advisors and Practice Facilitators* covers essential information and techniques to help primary care practices use electronic health records and other health IT for quality improvement (QI). This resource features information on clinical decision support, patient portals and other technologies, using patient-generated data, clinical quality measures, and risk stratification in primary care. The handbook includes helpful tips, examples, and use cases to support the use of health IT for QI efforts and is a complementary resource to AHRQ's Primary Care Practice Facilitation Curriculum and training modules.

SBIRT Oregon Screening tools: https://www.sbirtoregon.org/screening-forms/

From SAMHSA website: FindTreatment.gov is updated annually from facility responses to SAMHSA's National Substance Use and Mental Health Services Survey.

From The National Institute on Alcohol Abuse and Alcoholism (NIAAA): video series called "Short Takes with NIAAA," featuring commentary by NIAAA experts. The series consists of

© 2024 Huffstetler AN et al. *JAMA Health Forum*.

social media-friendly, 60-second videos explaining commonly used—but often misunderstood—alcohol terms.

The first installment of Short Takes provides concise, plain-language explanations of the following topics:

1. Alcohol overdose (also available in Spanish)
2. Alcohol use disorder
3. Binge drinking
4. Blackouts

© 2024 Huffstetler AN et al. *JAMA Health Forum*.

Practice Intake Assessment

We want to help primary care and research throughout Virginia. To help us do this, can you please tell us a little about your practice by answering the following questions and let us know how to may be able to contact you? We promise to be respectful with communications and we will not share your practice information or contact information. We appreciate you taking the time to complete this.

1. Practice ID [we generate]
2. Practice Name: ______________________________
3. Practice address: ______________________________
   City________________ State:____________ Zip Code: __________
4. Practice phone number: ______________________________
5. Practice fax number: ______________________________
6. Medical group name: ______________________________
7. Health system affiliation: ______________________________
8. Practice ownership:
   □ Hospital/health system □ Clinician owner □ Clinician partially owned
   □ Private sponsor/corporation □ Insurance company □ University owned
   □ Federally qualified health center □ Non-federal government clinic
   □ Military, Veterans administration, department of defense □ Health maintenance organization
   □ Indian Health Service
9. Is your practice recognized as a Patient Centered Medical Home (PCMH)? □ Yes □ No
10. Is your practice part of an Accountable Care Organization (ACO)? □ Yes □ No
    a. If you are part of an ACO, what is the name? ______________________
11. What best describes your type of practice? □ Primary care practice
    □ Specialty practice
    □ Mixed between primary care and specialty practice
12. Does your practice provide direct primary care (DPC) or charge your patients a monthly membership fee or concierge care? □ Yes, for all patients □ Yes, for some patients □ No
13. Describe the community your practice serves: □ Urban □ Suburban □ Rural
14. Best point of contact's name: ______________________________
15. Contact's role: □ Office manager □ Administrative personnel □ Clinician □ Nurse
16. Contact's email: ______________________________

© 2024 Huffstetler AN et al. *JAMA Health Forum*.

17. Contact's office phone:________________________________ext. #__________________________
18. Contact's cell phone:____☐ Prefer not to provide it
19. Contact's preferred method of communication: ☐ Mail ☐ Email ☐ Cell phone ☐ Work phone
20. Second point of contact's name: ____
21. Second contact's role: ☐ Office manager ☐ Administrative personnel ☐ Clinician ☐ Nurse
    ☐ Other ______________________
22. Second contact's email: ____
23. Second contact's office phone:________________________ext.# __________________________
24. Second contact's cell phone:____________________________________☐ Prefer not to provide it
25. Second contact's preferred method of communication:
    ☐ Mail ☐ Email ☐ Cell phone ☐ Work phone
26. Can we send you periodic texts about research (less than once per month)? ☐ Yes ☐ No
    a. If yes, what is the best number to text? ____

27. In one full day of patient care, on average, how many patients does a clinician in your practice see? ____

28. Do clinicians in your practice have their own panel of patients that they are responsible for?
    ☐ Yes ☐ No
    a. If yes, please estimate the average patient panel size for a full-time clinician in your practice: ____

29. What EMR does your practice use? ____
    29a. ☐ N/A, we use paper charts
30. Does your practice regularly teach students? ☐ Yes ☐ No
31. Does your practice regularly teach residents? ☐ Yes ☐ No
32. Do clinicians from your practice provide inpatient care when patients from the practice are admitted to the hospital?
    ☐ Yes, clinicians provide inpatient care
    ☐ No, clinicians visit patients in hospital but do not provide inpatient care
    ☐ No, hospital-based staff provides all care for inpatients

33. Does any clinician in your practice provide care in a language other than English? ☐ Yes ☐ No
    a. If yes, what are the other languages? ____

© 2024 Huffstetler AN et al. *JAMA Health Forum*.

34. Is your practice currently accepting new patients with the following insurance types? *Please check one appropriate box for each insurance type*.

	Yes, ALL new patients	Yes, MOST new patients	Yes, SOME new patients	No, not accepting any patients
Commercial/Private				
Self-pay				
Medicaid				
Medicare				
Uninsured				

35. Thinking about your practice specifically, how would you describe the competitive situation your practice faces?

□ Very competitive □ Somewhat competitive □ Not at all competitive

36. Please indicate if your practice has experienced any of the following major changes in the past 12 months?

a. Moved to a new office? □ Yes □ No
b. New medical records system? □ Yes □ No
c. Changed ownership? □ Yes □ No
d. Had office renovations? □ Yes □ No
e. New billing system? □ Yes □ No
f. Significant clinician turnover? □ Yes □ No
g. Any other major change? ____

37. Please indicate if you anticipate that your practice will experience any of the following major changes in the next 12 months?

a. Move to a new office? □ Yes □ No
b. New medical records system? □ Yes □ No
c. Change in ownership? □ Yes □ No
d. Office renovations? □ Yes □ No
e. New billing system? □ Yes □ No
f. Significant clinician turnover? □ Yes □ No
g. Any other major change? ____

© 2024 Huffstetler AN et al. *JAMA Health Forum*.

38. What services does your practice provide?
    a. Same day appointments? □ Yes □ No
    b. 24-hour telephone triage? □ Yes □ No
    c. Evening appointments? □ Yes □ No
    d. Weekend appointments? □ Yes □ No
    e. Patient portal? □ Yes □ No
    f. Telehealth visits? □ Yes □ No
    g. Group visits? □ Yes □ No
    h. Onsite lab? □ Yes □ No
    i. Onsite radiology? □ Yes □ No
    j. Patient navigation? □ Yes □ No
    k. Care coordination? □ Yes □ No

39. What population health strategies does your practice use?
    a. Use of a registry or list to identify patients in need of care? □ Yes □ No
    b. Routine measurement of quality or performance? □ Yes □ No
    c. Partnership with community or health system programs to address unhealthy behaviors? □ Yes □ No
    d. Partnership with community or health system programs to address mental health needs? □ Yes □ No
    e. Partnership with community or health system programs to address social needs? □ Yes □ No

40. What are the biggest challenges that your practice currently faces?

____

____

41. ~~Would you be interested in participating in research that matters to primary care? □ Yes □ No~~

<u>Patients</u>

42. Please provide your best *estimate* for what your practice's current payer mix is. *Total should add up to 100%.*

	Percent (%)
Commercial/Private	
Medicaid	
Medicare	
Dual Medicaid and Medicare	
Self-Pay/Uninsured	

© 2024 Huffstetler AN et al. *JAMA Health Forum*.

43. Please provide your best *estimate* for what percentage of your patients belong to each of the following racial categories. *Total should add up to 100%.*

	Percent (%)
White	
African-American/Black	
American Indian or Alaskan Native	
Asian	
Native Hawaiian or other Pacific Islander	
Other	

44. Please provide your best *estimate* for what percentage of your patients belong to each of the following ethnic categories. *Total should add up to 100%.*

	Percent (%)
Hispanic or Latino	
Non-Hispanic or Non-Latino	

45. Please provide your best *estimate* for what your percentage of your patients belong to each of the following age categories. *Total should add up to 100%.*

	Percent (%)
0-17 years	
18-39 years	
40-59 years	
60-75 years	
76 years and over	

46. Please provide your best *estimate* for what percentage of your patients belong to each of the following gender categories. *Total should add up to 100%.*

	Percent (%)
Men, including transgender men	
Women, including transgender women	
Non-binary, gender non-conforming, third gender, or gender fluid	
Prefer not to self-describe	

Name of person completing survey: ___________________________________

Who did you consult to complete this survey?

© 2024 Huffstetler AN et al. *JAMA Health Forum*.

Practice Team Survey

Workflow for screening for unhealthy alcohol use

1. The practice has implemented a process for screening patients for the frequency and quantity of alcohol use with a **validated questionnaire** (such as the AUDIT-C, and SASQ).
    a. Not started at this time (skip to question 2)
    b. Just beginning to implement
    c. Actively addressing, almost completed
    d. Fully implemented across the practice

    If the response is b, c, or d:
    a. What questions are used?
    b. Which patients are screened?
        i. All patients ages 18 and above?
        ii. All patient with different age range (specify___________________)
        iii. Patients with a particular condition (specify___________________)
        iv. Patients presenting for a health maintenance visit
        v. Other (specify____)
    c. Is it done by…
        a. A few clinicians
        b. Some clinicians
        c. Most clinicians
        d. All clinicians
    d. Who administers the screening questions? (e.g. rooming staff, clinician)

    e. When in the workflow are the screening questions administered? (e.g. at check-in, at conclusion of rooming process, during discussion with clinician)

    f. How are they administered (e.g. verbally, by paper, electronically)?

    g. How often are patients screened?
        vi. Every visit
        vii. Annually
        viii. Once
        ix. Other (specify_________________________)

2. The practice has implemented a workflow for reviewing and interpreting alcohol screening results.
    a. Not started at this time
    b. Just beginning to implement
    c. Actively addressing, almost completed
    d. Fully implemented across the practice

    If so, please describe who completes this review and interpretation (e.g. nurses, clinicians, behavioral health providers).

© 2024 Huffstetler AN et al. *JAMA Health Forum*.

3. The practice has implemented a process for further assessment among patients with a positive screening result to determine their level of unhealthy alcohol use.
   a. Not started at this time
   b. Just beginning to implement
   c. Actively addressing, almost completed
   d. Fully implemented across the practice

   If the response is b, c, or d: What assessment instrument do they use (examples: AUDIT, Alcohol Symptom Checklist)? _________________

Workflow for counseling for unhealthy alcohol use

4. The practice has implemented a process for routinely providing feedback to patients on their screening results and initial counseling to decrease use or stop drinking alcohol when indicated.
   a. Not started at this time
   b. Just beginning to implement
   c. Actively addressing, almost completed
   d. Fully implemented across the practice

5. The practice has implemented a system for continued counseling, referral, and coordination of care for patients with unhealthy alcohol use and alcohol use disorder.
   a. Not started at this time
   b. Just beginning to implement
   c. Actively addressing, almost completed
   d. Fully implemented across the practice

6. The practice provides the following support to help patients with unhealthy drinking and alcohol use disorder:

☐ Counseling by clinician	☐ Help patients with stress management
☐ Counseling by a behavioral therapist	☐ Provide patients with community resources
☐ Motivational interviewing	☐ Refer to a behavioral therapist
☐ Provide patients with educational materials	☐ Refer to outpatient programs (AA, CSB)
☐ Encourage drinking diaries	☐ Refer to inpatient programs
☐ Help patients make action plans	☐ Prescribe medication assistance therapy
☐ Give alcohol use prescriptions	☐ Refer or provide group therapy
☐ Help patients build a support network	☐ Other services
☐ Help patients identify pitfalls	

   For the above items, are they provided by (note if different for different items):
   a. A few clinicians
   b. Some clinicians
   c. Most clinicians
   d. All clinicians

© 2024 Huffstetler AN et al. *JAMA Health Forum*.

7. The practice has implemented a structured system to assure follow-up with patients receiving treatment for unhealthy alcohol use and alcohol use disorders (may include phone calls, visits, telehealth).
    a. Not started at this time
    b. Just beginning to implement
    c. Actively addressing, almost completed
    d. Fully implemented across the practice

Implementation Climate Scale

Instructions: Please indicate the extent to which you agree with each statement.

0<br>Not at all	1<br>Slight extent	2<br>Moderate extent	3<br>Great extent	4<br>Very great extent

Focus on Evidence-Based Practice

1. One of this team/agency's main goals is to use evidence-based practices effectively.....	0 1 2 3 4
2. People in this team/agency think that the implementation of evidence-based practices is important..	0 1 2 3 4
3. Using evidence-based practices is a top priority in this team/agency............................	0 1 2 3 4

Educational Support for Evidence-based Practice

4. This team/agency provides conferences, workshops, or seminars focusing on evidence-based practices...	0 1 2 3 4
5. This team/agency provides evidence-based practice trainings or in-services................	0 1 2 3 4
6. This team/agency provides evidence-based practice training materials, journals, etc....	0 1 2 3 4

Recognition for Evidence-Based Practice

7. Clinicians in this team/agency who use evidence-based practices are seen as clinical experts...	0 1 2 3 4
8. Clinicians who use evidence-based practices are held in high esteem in this team/agency...	0 1 2 3 4
9. Clinicians in this team/agency who use evidence-based practices are more likely to be promoted..	0 1 2 3 4

© 2024 Huffstetler AN et al. *JAMA Health Forum*.

Rewards for Evidence-Based Practice

10. This team/agency provides financial incentives for the use of evidence-based practices 0 1 2 3 4

11. The better you are at using evidence-based practices, the more likely you are to get a bonus or a raise........ 0 1 2 3 4

12. This team/agency provides the ability to accumulate compensated time for the use of evidence-based practices........ 0 1 2 3 4

Selection for Evidence-Based Practice

13. This team/agency selects staff who have previously used evidence-based practice....... 0 1 2 3 4

14. This team/agency selects staff who have had formal education supporting evidence-based practice 0 1 2 3 4

15. This team/agency selects staff who value evidence-based practice........ 0 1 2 3 4

Selection for Openness

16. This team/agency selects staff who are adaptable........ 0 1 2 3 4

17. This team/agency selects staff who are flexible........ 0 1 2 3 4

18. This team/agency selects staff open to new types of interventions 0 1 2 3 4

© 2024 Huffstetler AN et al. *JAMA Health Forum*.

eAppendix 2. Sensitivity Analysis – Screening, Counseling, and Treatment Outcomes for All Patients Aged 18 to 79 Years with a Visit Eligible for Screening

This analysis assessed practice as randomized to early or delayed intervention. Accordingly, two practices randomized to early intervention, but who received the delayed intervention, were analyzed as if they received the early intervention; and two practices randomized to delayed intervention, but who received the early intervention, were analyzed as if they received the delayed intervention.

		Baseline	Three Months	Six Months
**Screening Results for All Patients Practices Determined Eligible (n=11,789)<br>Adjusted percentage (95% Confidence Interval)**				
Documentation of alcohol use [a]	Intervention	0.717 (0.598 – 0.812)	0.739 (0.624 – 0.829)	0.827 (0.736 – 0.891)
	Control	0.798 (0.076 – 0.866)	0.712 (0.602 – 0.802)	0.716 (0.606 – 0.805)
ICC = 0.336	p-value [b]	--	--	<.001 (t = 9.63)
Screening with AUDIT-C or SASQ	Intervention	0.023 (0.005 – 0.095)	0.060 (0.014 – 0.220)	0.291 (0.086 – 0.643)
	Control	0.003 (0.001 – 0.015)	0.009 (0.002 – 0.038)	0.032 (0.008 – 0.123)
ICC = 0.781	p-value [b]	--	--	0.076 (t = 1.77)
Unhealthy alcohol use identified	Intervention	0.048 (0.033 – 0.069)	0.058 (0.040 – 0.083)	0.066 (0.046 – 0.093)
	Control	0.043 (0.030 – 0.061)	0.045 (0.032 – 0.064)	0.054 (0.038 – 0.075)
ICC = 0.123	p-value [b]	--	--	0.606 (t = 0.52)
**Counseling and Treatment Results for All Patients Who Screened Positive or Had a Diagnosis of UAU or AUD (n=895)<br>Adjusted percentage (95% Confidence Interval)**				
Brief office intervention	Intervention	0.318 (0.170 – 0.514)	0.584 (0.387 – 0.757)	0.646 (0.455 – 0.800)
	Control	0.416 (0.250 – 0.604)	0.511 (0.331 – 0.689)	0.532 (0.347 – 0.708)
ICC = 0.309	p-value [b]	--	--	0.038 (t = 2.08)
Referral for counseling and treatment	Intervention	0.307 (0.164 – 0.501)	0.567 (0.372 – 0.743)	0.632 (0.442, 0.789)
	Control	0.405 (0.243 – 0.591)	0.513 (0.334 – 0.689)	0.540 (0.356 – 0.713)
ICC = 0.290	p-value [b]	--	--	0.063 (t = 1.87)
Medication for alcohol use disorder	Intervention	0.018 (0.005 – 0.069)	0.051 (0.016 – 0.151)	0.047 (0.015 – 0.134)
	Control	0.012 (0.003 – 0.045)	0.021 (0.006 – 0.071)	0.060 (0.021 – 0.158)

© 2024 Huffstetler AN et al. *JAMA Health Forum*.

ICC = 0.253	p-value [b]	--	--	0.410 (t = -0.82)
Any intervention or treatment	Intervention	0.005 (0.001 – 0.048)	0.014 (0.002 – 0.091)	0.017 (0.003 – 0.091)
	Control	0.019 (0.003 – 0.010)	0.023 (0.004 – 0.109)	0.024 (0.005 – 0.114)
ICC = 0.313	p-value [b]	--	--	0.475 (t = 0.71)

a – any documentation of alcohol use, includes non-structured notations in text about alcohol use, use of recommended screening instruments, and diagnosis of UAU or AUD.

b – p values compare the difference in differences between 6 months and the baseline for the intervention and control groups.

UAU = unhealthy alcohol use, AUD = alcohol use disorder, ICC = Intraclass Correlation

As expected, these results were attenuated compared to analyzing practices based on the intervention practices actually received. Specifically, in the intention to treat analysis, the 2 delayed intervention practices serving as controls who received the intervention made increases in screening and brief counseling rates. The opposite is true for the 2 early intervention practices that actually did not receive the intervention in the 6-month observation period.

The general trends for all findings remain similar. For screening with the AUDIT-C or SAS-Q the p value became nonsignificant (p=0.076), but the p values for all other findings (increased documentation in alcohol use and brief office intervention remained significant).

Comparison of Significant Findings for Analysis Based on Intervention Received versus Intention to Treat Analysis

	**Analysis Based on Intervention Received**		**Analysis Based on Intervention Randomized to (Intention to Treat)**	
	**Change from Baseline to 6 months**	**P value**	**Change from Baseline to 6 months**	**P value**
**Documentation of alcohol use**				
Intervention	7.5%	<0.001	11.0%	<0.001
Control	-5.3%		-0.8%	
**Screening with AUDIT-C or SASQ**				
Intervention	33.4%	<0.001	26.8%	0.076

© 2024 Huffstetler AN et al. *JAMA Health Forum*.

Control	1.0%		2.9%	

**Brief office intervention**				
Intervention	36.4%	0.008	32.8%	0.038
Control	9.6%		11.6%	

© 2024 Huffstetler AN et al. *JAMA Health Forum*.
