# Supplementary material for: Practice Facilitation to Address Unhealthy Alcohol Use in Primary Care: A Cluster Randomized Clinical Trial
Source: JAMA Health Forum. 2024 Aug 9;5(8):e242371. doi: 10.1001/jamahealthforum.2024.2371 (PMC11316228; doi:10.1001/jamahealthforum.2024.2371)
Supplement: Supplement 3. — Data Sharing Statement [file jamahealthforum-e242371-s003.pdf]

Data Sharing Statement

Huffstetler. Practice Facilitation to Address Unhealthy Alcohol Use in Primary Care. *JAMA Health Forum*. Published August 09, 2024. doi:10.1001/jamahealthforum.2024.2371

Data
Data available: Yes
Data types: Data (not involving human participants), Data dictionary, Other (please specify)
Additional Information: Study data may be made available in aggregate upon request of authors
How to access data: alexander.krist@vcuhealth.org
When available: With publication

Supporting Documents
Document types: Statistical/analytic code
How to access documents: Analytic code may be made available in aggregate upon request of authors
When available: With publication

Additional Information
Who can access the data: Anyone requesting data with a proposed analysis and approved protocol.
Types of analyses: To inform research, policy, and clinical care
Mechanisms of data availability: After approval of a proposal
Any additional restrictions: None
